# Supplementary material for: Deep learning–integrated multilayer thermal gradient sensing platform for real-time blood flow monitoring
Source: Sci Adv. 2026 Feb 6;12(6):eaea8902. doi: 10.1126/sciadv.aea8902 (PMC12880533; doi:10.1126/sciadv.aea8902)
Supplement: 20260206-1 [file sciadv.aea8902.v1.pdf]

ENGINEERING

Deep learning–integrated multilayer thermal gradient sensing platform for real-time blood flow monitoring

Youngmin Sim[1], Yosep Park[1], Kyeongha Kwon[1,2]*

Copyright © 2026 The Authors, some rights reserved; exclusive licensee American Association for the Advancement of Science. No claim to original U.S. Government Works. Distributed under a Creative Commons Attribution License 4.0 (CC BY).

Blood flow monitoring is fundamental for assessing cardiovascular health and identifying vascular complications. Traditional Doppler ultrasound methods require bulky equipment and specialized expertise, while recent thermal sensing approaches face limitations due to blood vessel depth variability beneath the skin. We present a soft electronic platform that integrates multilayer thermal sensing with deep learning algorithms to simultaneously measure blood flow rate and vessel depth. The device uses a wireless system with thermal sensing modules, featuring strategically positioned thermistors in separate layers to capture thermal gradients at different heights from the skin surface. Deep learning processes multilayer thermal patterns to extract both parameters in real time. Validation through benchtop testing, finite element analysis, and on-body trials demonstrates accurate measurements across relevant flow rates and vessel depths. Integration with photoplethysmography enhances continuous blood pressure monitoring accuracy compared to conventional approaches, particularly during dynamic physiological changes. This technology offers potential for personalized cardiovascular monitoring, early detection of hemodynamic events, and skin graft surveillance.

INTRODUCTION

Blood flow plays critical roles within the human body, delivering oxygen, nutrients, hormones, and waste products and maintaining homeostasis and health (*1*–*5*). Blood flow monitoring can provide valuable insights for diagnosing basic health states and identifying potential vascular damages (*6*–*8*). Blood flow rates are typically measured using a Doppler ultrasound technique, which requires bulky medical machines and professional knowledge (*9*–*11*). Recent studies have demonstrated miniaturized, wearable platforms that measure flow rates in microfluidic channels using thermal sensing with thermistors and actuators (*12*, *13*). These devices detect flow rates by measuring temperature differences between upstream and downstream thermistors. While thermal sensing methods are effective for microfluidic applications where the channel depth is fixed and known, they face substantial limitations when applied to blood flow measurement due to the inherent variability in vessel depth beneath the skin. This variability introduces a critical measurement challenge, as the temperature difference detected by thermistors is influenced by the blood flow rate and the vessel depth. Conventional single-layer thermal sensors cannot differentiate between these two variables, making it difficult to obtain accurate flow measurements without prior knowledge of vessel depth. Existing thermal sensing approaches for blood flow have claimed discrete depth classifications capability (*11*) or have demonstrated qualitative flow status detection (patent, low, and occluded) (*13*), but none provide simultaneous continuous quantification of both flow rate and vessel depth parameters (see table S1 for details).

Blood flow rate and vessel depth modulate heat distribution patterns, manifesting in lateral and vertical thermal gradients relative to the skin surface. Therefore, temperature distributions measured at elevated positions from the skin surface may provide quantitative information regarding vessel depth. Here, we leverage this principle by developing a skin-interfaced platform that measures temperature distributions at multiple elevated positions to wirelessly monitor blood flow rate and vessel depth in real time. The platform uses a multilayer thermal sensing module (TSM) with strategically placed thermistors in separate layers, enabling simultaneous measurement of thermal gradients at different heights from the skin surface. When interfaced with the skin, the device's layered architecture allows for comprehensive thermal profiling across various vessel depths. These thermal maps reveal distinctive spatial temperature patterns for each sensing layer, providing complementary information that cannot be obtained from single-layer measurements. A wireless system integrating the TSM with a Bluetooth low energy (BLE) system on a chip (SoC) transmits the acquired thermal data to a user interface (UI), where deep learning algorithms process these thermal patterns to extract both vessel depth and flow rate parameters in real time (20 Hz). This approach provides continuous (1 Hz) measurements across the full spectrum of physiologically relevant blood flow rates (1 to 10 mm/s) and vessel depths (1 to 2 mm) (*11*). The thermal sensing approach captures time-averaged blood flow velocity rather than instantaneous pulsatile variations. The effectiveness of this approach is demonstrated through comprehensive benchtop testing, finite element analysis (FEA) simulations of thermal propagation patterns, and on-body trials with healthy participants under various physiological conditions. Our multilayer thermal sensor achieves robust blood flow measurement accuracy of ±0.12 mm with ±0.07-mm depth resolution through simultaneous, continuous quantification of flow and vessel depth in a wireless format that eliminates the tethering constraints of existing wired systems (*11*, *13*). In addition, combining our blood flow measurements with photoplethysmography (PPG) signals reduces continuous blood pressure estimation error by 9.54 to 72.6% compared to the PPG-only method. Existing continuous blood pressure monitoring systems require either specialized equipment (*14*) providing beat-to-beat measurements but limiting patient mobility or portable ambulatory monitors (*15*, *16*) that operate intermittently at 15- to 60-min intervals. Furthermore, both types rely on cuff-based measurements,

[1]School of Electrical Engineering, Korea Advanced Institute of Science and Technology, Daejeon, Republic of Korea. [2]Graduate School of Artificial Intelligence Semiconductor, Korea Advanced Institute of Science and Technology, Daejeon, Republic of Korea.
*Corresponding author. Email: kyeongha@kaist.ac.kr

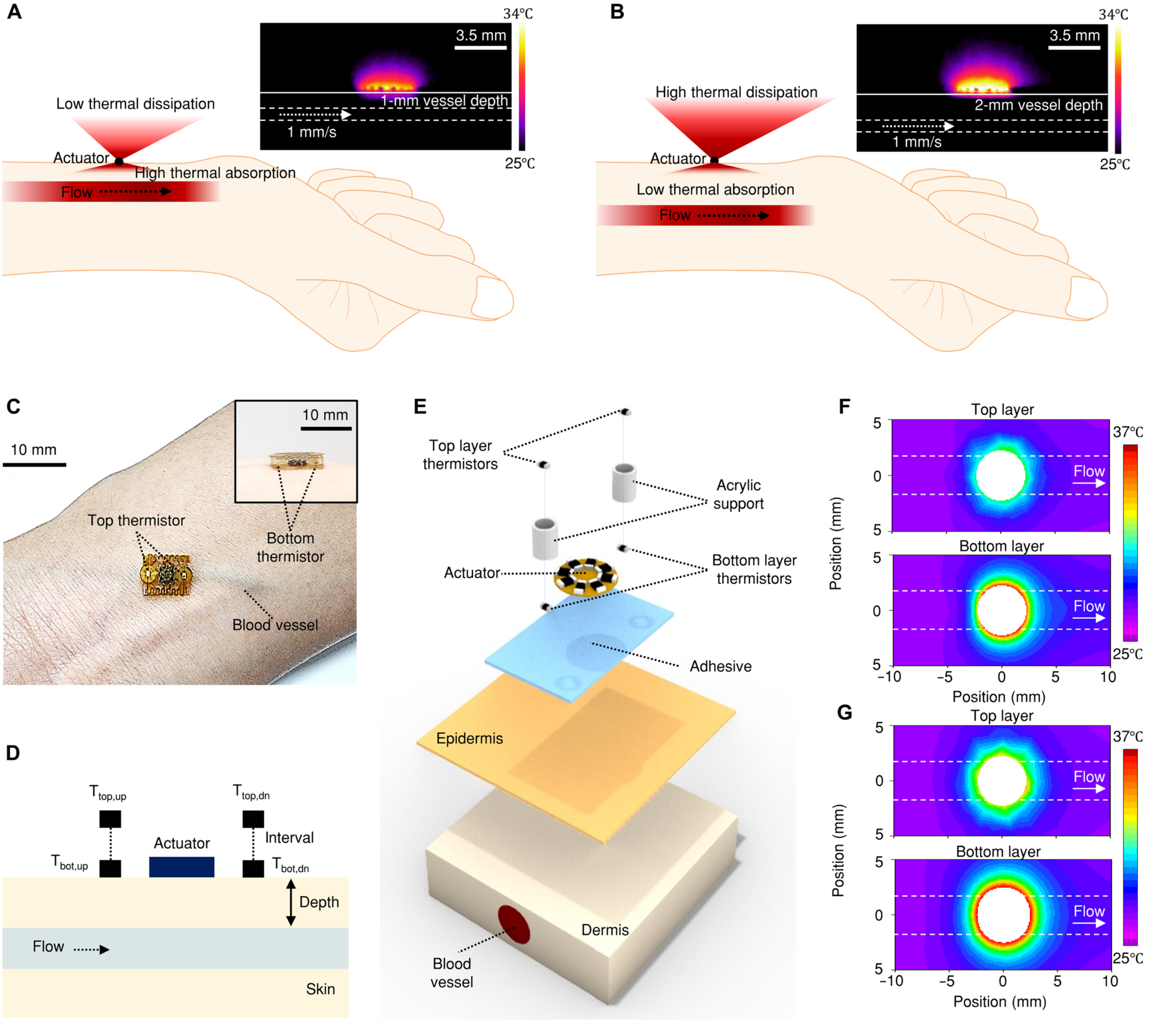

Fig. 1. Multilayer thermal sensing architecture for simultaneous blood flow and vessel depth monitoring. (**A** and **B**) IR thermal imaging of a 3.5-mm diameter thermal actuator positioned over blood vessel phantoms at depths of 1 mm (A) and 2 mm (B) from the skin surface, demonstrating depth-dependent absorption and dissipation patterns. Flow velocity maintains 1 mm/s with thermal power density of 5.89 mW/mm^{-2}. (**C**) Photograph of the multilayer thermal sensor over a wrist vein. Inset provides a side view. (**D**) Cross-sectional schematic illustrating the spatial arrangement of upstream ($T_{top,up}$ and $T_{bot,up}$) and downstream ($T_{top,dn}$ and $T_{bot,dn}$) thermistors in both top/bottom sensing layers relative to the central thermal actuator and blood vessel orientation. (**E**) Exploded-view illustration of the sensor architecture showing the layered assembly: top and bottom thermistor arrays, acrylic support structures maintaining 1.5-mm interlayer spacing, thermal actuator, and adhesives. (**F** and **G**) FEA results showing planar temperature distributions in top and bottom sensing layers for vessel depths of 1 mm (F) and 2 mm (G) with 1-mm/s flow rate. Temperature ranges from 25° to 37°C.

posing challenges for neonates and patients with arm injuries where conventional cuff placement is impractical. While PPG-based methods (*17–19*) offer cuff-free blood pressure estimation, they suffer from inadequate tracking of rapid hemodynamic fluctuations. Our integrated approach enables wearable, continuous (1 Hz) blood pressure monitoring without cuff-based constraints, yielding reduced 9.54 to 72.6% error reduction and better agreement with clinical-grade monitors, particularly during physiological changes where traditional methods show degraded performance.

RESULTS

Multilayer thermal gradient sensor

The thermal response to cutaneous actuation is affected by vascular depth, wherein deeper vessels exhibit lower absorption and higher dissipation, resulting in more diffused thermal patterns due to increased lateral heat dissipation through tissue (*11*). To visualize the thermal physics, the infrared (IR) images in Fig. 1 (A and B) show the lateral-view measurements of skin phantoms designed to model blood vessels (2.5-mm diameter) at depths of 1 and 2 mm, respectively, from the

skin surface (for details, see Materials and Methods and fig. S1). The thermal actuator (3.5-mm diameter) generates constant thermal power density (P_d = 5.89 mW mm^{-2}), delivering heat to the flowing liquid below (1 mm/s). The resulting heat patterns vary depending on vessel depth, thereby necessitating multilayer sensor configurations for effective noninvasive blood flow monitoring.

Figure 1C shows an optical image of the sensor placed on the skin over a blood vessel. The inset shows the tilted side view. The sensor comprises a thermal actuator, two pairs of upstream-downstream thermistors positioned in top and bottom sensing layers ($T_{top,up}$, $T_{top,dn}$, $T_{bot,up}$, and $T_{bot,dn}$; Fig. 1D), two acrylic supports (upstream/downstream) that maintain a fixed interval between top/bottom layers, and an adhesive interface for skin attachment (Fig. 1E). The thermal actuator consists of eight 24-Ω resistors connected in series, through which constant current is applied to provide controlled thermal excitation to the underlying tissue. Thermistors with dimensions of 0.3 mm by 0.6 mm are strategically positioned 3.5 mm from the center of the actuator in both the top and bottom sensing layers. See Materials and Methods and fig. S2 for more details. The spacing between these layers is maintained at 1.5 mm, preserving the wearability of the device while still providing sufficient separation to capture distinct thermal profiles at different vessel depths. This multilayer sensor captures thermal signatures at different elevations from the skin surface, allowing simultaneous determination of vessel depth and blood flow rate.

FEA results in Fig. 1 (F and G) show planar temperature distributions in the top and bottom layers for a blood flow rate of 1 mm/s at vessel depths of 1 and 2 mm, respectively (for details, see fig. S3 and Materials and Methods). The temperature ranges from 25° to 37°C across the sensing area. Upon thermal actuation, vessels at 1-mm depth exhibit greater heat absorption with concentrated thermal patterns, while vessels at 2-mm depth show reduced absorption with wider temperature distributions due to increased outward dissipation. Both scenarios show characteristic downstream skewing in the flow direction. This combination of flow-dependent skewing and depth-dependent absorption enables the simultaneous determination of vessel depth and flow rate.

Wireless system with deep learning inference

Figure 2A shows the optical image (top view) of the wireless blood flow sensing module with BLE communication capabilities. The device dimensions are 27.8 mm by 27.2 mm by 1.5 mm (width by length by thickness), with a total mass of 425 mg (excluding battery). The inset shows the side view of the sensor, highlighting the 1.5-mm separation between top/bottom sensing layers achieved via acrylic supports (see Materials and Methods and fig. S4 for details). Figure S5 presents an exploded-view illustration of the wireless platform. Figure 2B highlights the electronic components for actuation and sensing on a flexible printed circuit board (fPCB) designed for folding into a multilayer sensing configuration, with a centrally positioned actuator and symmetrically arranged thermistors in both layers. Figure 2C shows an IR image of the device placed over a vein using bench power supply, revealing temperature gradients from 30° to 38°C. The thermal patterns in the IR image demonstrate heat propagation along the direction of blood flow, creating an asymmetric temperature distribution that shifts downstream. Concurrent monitoring confirmed that skin surface temperature rise remained <3°C above baseline during operation, and no visible irritation was observed after >30 min of continuous wear.

Figure 2D illustrates the schematic diagram of the platform, which comprises a TSM integrated with a BLE SoC. The TSM includes a two-layered blood flow sensor (Fig. 1) with additional two-layered reference thermistors ($TH_{top,ref}$ and $TH_{bot,ref}$). The top layer uses three thermistors ($TH_{top,up}$, $TH_{top,dn}$, and $TH_{top,ref}$) arranged in Wheatstone-bridge configurations with fixed resistors R, followed by differential amplifiers. Bottom layer thermistors ($TH_{bot,up}$, $TH_{bot,dn}$, and $TH_{bot,ref}$) have an identical configuration. These amplifiers compare upstream and downstream thermistor readings against a reference thermistor positioned at equal distances from the actuator but outside the flow path. This arrangement effectively eliminates environmental temperature variations, isolating signals specific to blood flow and vessel depth.

Upon measurement initiation, the BLE SoC activates general-purpose input/output (GPIO) pins to deliver a constant current (17.2 mA) to the resistive heater (8 × 24 Ω). The resulting TSM output signals are converted to digital format through analog-to-digital converters. The processing unit applies a moving average filter with a smoothing coefficient of 0.05 to these signals, reducing high-frequency noise while preserving essential thermal signature patterns. The filtered data are then transmitted via the BLE radio to a smartphone for further processing. The smartphone application uses deep learning algorithms implemented through a neural network to analyze multilayer thermal patterns, simultaneously determining blood flow rate and vessel depth in real time (for details, see fig. S6 and movie S1). The neural network (Fig. 2E) accepts six inputs (upstream and downstream thermistor inputs from each sensing layer and two reference thermistor inputs) to produce two outputs (flow rate and vessel depth). The architecture features linear layers with rectified linear unit (ReLU) activation functions, batch normalization, and 10% dropout regulation optimized for real-time thermal pattern processing (see text S1 for more details).

System performance characterization and optimization

Sensor design parameters include (i) actuator diameter (D) and heating power (P) to control thermal input, and (ii) actuator-thermistor distance (L) and interlayer spacing (H) to define the measurement geometry (Fig. 3A). To optimize these parameters, benchtop studies used a microfluidic device with artificial vascular channels (fig. S7) and a syringe pump generating precisely controlled flow rates (f_{SET}) at room temperature [(RT); for details, see Materials and Methods and fig. S8]. This setup enabled systematic evaluation of root mean square error ($RMSE_f$) of flow sensing measured by the prototype (f_{MEAS}), $RMSE_f = \sqrt{\sum_{i=1}^{n}\left(f_{SET,i} - f_{MEAS,i}\right)^2 / n}$, across various parameter combinations (D, P, L, and H).

Figure 3B shows the $RMSE_f$ values (n = 1500) for various heat source profiles, with D of 2, 3.5, and 5.6 mm and P of 37.8, 56.7, and 75.6 mW (L = 3.5 mm, H = 1.5 mm). For each combination of D and P values, the $RMSE_f$ value was calculated using fivefold cross-validation (*20*) on 1500 measurement points. Measurements were collected across 30 experimental conditions, varying vessel depths (d_{SET}) of 1, 1.5, and 2 mm and flow rates (f_{SET}) from 1 to 10 mm/s (1 mm/s increments), with 50 measurements per condition (total n = 1500). K-fold cross-validation is a widely adopted technique that partitions data into K subsets, iteratively using K-1 subsets for training and one for testing, thereby ensuring that all data contribute to both training and evaluation phases. This approach mitigates the dependency on a single train-test split and provides more stable performance assessment, making it particularly valuable for evaluating

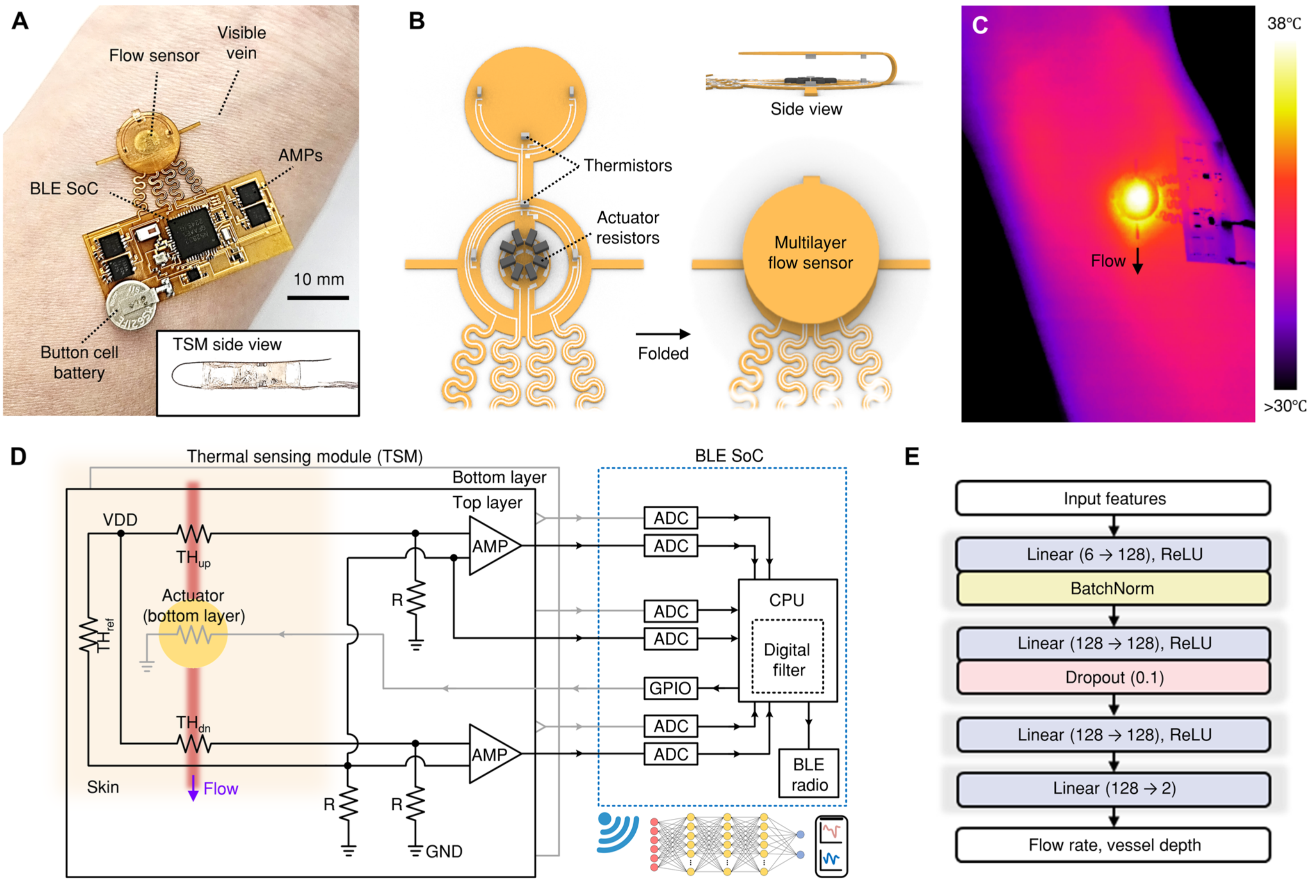

Fig. 2. Wireless blood flow sensing platform with deep learning integration. (**A**) Photograph of the flexible blood flow sensing module with BLE communication capabilities. The width, length, and thickness of the device are 27.8, 27.2, and 1.5 mm, respectively, excluding battery. Inset shows side view illustrating the 1.5-mm separation between sensing layers through acrylic support. (**B**) Schematic exploded view of the multilayer flow sensor showing the fPCB designed for folding configuration. (**C**) IR thermal image showing temperature gradients (30° to 38°C) and asymmetric heat propagation along blood flow direction. (**D**) Circuit and block diagram of the platform architecture comprising the TSM integrated with BLE SoC. The TSM consists of two-layered upstream (TH_{up}), downstream (TH_{dn}), and reference (TH_{ref}) thermistors with Wheatstone bridge configurations, differential amplifiers (AMPs), variable gain amplifiers, and analog-to-digital converters (ADCs). VDD, supply voltage; GND, ground. (**E**) Deep learning neural network architecture accepting six input features (upstream, downstream, reference thermistor inputs from each sensing layer) to generate two outputs: flow rate and vessel depth. The network uses linear layers with ReLU activation, batch normalization (BatchNorm), and 10% dropout regularization.

sensor systems with limited experimental datasets. Here, for fivefold cross-validation, the 30 experimental conditions were grouped into five subsets of six conditions each, with four subsets (4 × 6 conditions × 50 measurements = 1200 measurements) used for training and one subset (1 × 6 conditions × 50 measurements = 300 measurements) for testing in each fold. This process was repeated five times to ensure that every condition contributed to testing, resulting in $RMSE_f$ calculation based on all 1500 measurements. The results show that the model generalizes to untrained flow-depth combinations by learning underlying thermal gradient patterns. As shown in Fig. 3B, increasing D and P that correspond to increasing thermal input reduces $RMSE_f$, although the performance improvements become marginal beyond $D \geq 3.5$ mm and $P \geq 56.7$ mW. Statistical analysis of the four top-performing configurations reveals overlapping 95% confidence intervals (RMSE: 0.1281 ± 0.0161, 0.1280 ± 0.0160, 0.1293 ± 0.0173, and 0.1286 ± 0.0166), indicating no statistically significant differences among these optimal parameter combinations.

Figure 3C shows the $RMSE_f$ results ($n = 1500$) for various measurement geometry configurations, with L of 2, 3.5, and 5.6 mm and H of 1, 1.5, and 2 mm ($D = 3.5$ mm, $P = 56.7$ mW). The cross-validation methodology remained consistent with that used for thermal input parameters. Despite optimal performance ($RMSE_f = 0.1171$) achieved at maximum dimensions (L = 5.6 mm, H = 2 mm), intermediate sensor dimensions (L = 3.5 mm and H = 1.5 mm) achieved a comparable $RMSE_f$ of 0.1293 while accommodating better alignment with the vessel trajectory beneath the sensor due to its smaller size.

Further studies (Fig. 3, D to G) validated devices with the selection of $D = 3.5$ mm, $P = 56.7$ mW, $L = 3.5$ mm, and $H = 1.5$ mm, on an expanded dataset of 9000 measurement points at RT (300 measurements for each combination of d = 1, 1.5, and 2 mm and f_{SET} = 1,2,3,…,10 mm/s). The neural network was trained using this optimized sensor configuration to predict flow rate and vessel depth parameters. Results in Fig. 3 (D and E) demonstrate linear correlations between f_{SET} and f_{MEAS} and between d_{SET} and vessel depth measured by the prototype (d_{MEAS}), respectively, with all individual data points plotted. Figure 3F shows the RMSE of d_{MEAS} ($RMSE_d = \sqrt{\sum_{i=1}^{n} \left(d_{SET,i} - d_{MEAS,i}\right)^2 / n}$; $n = 900$), remaining below 0.07 mm

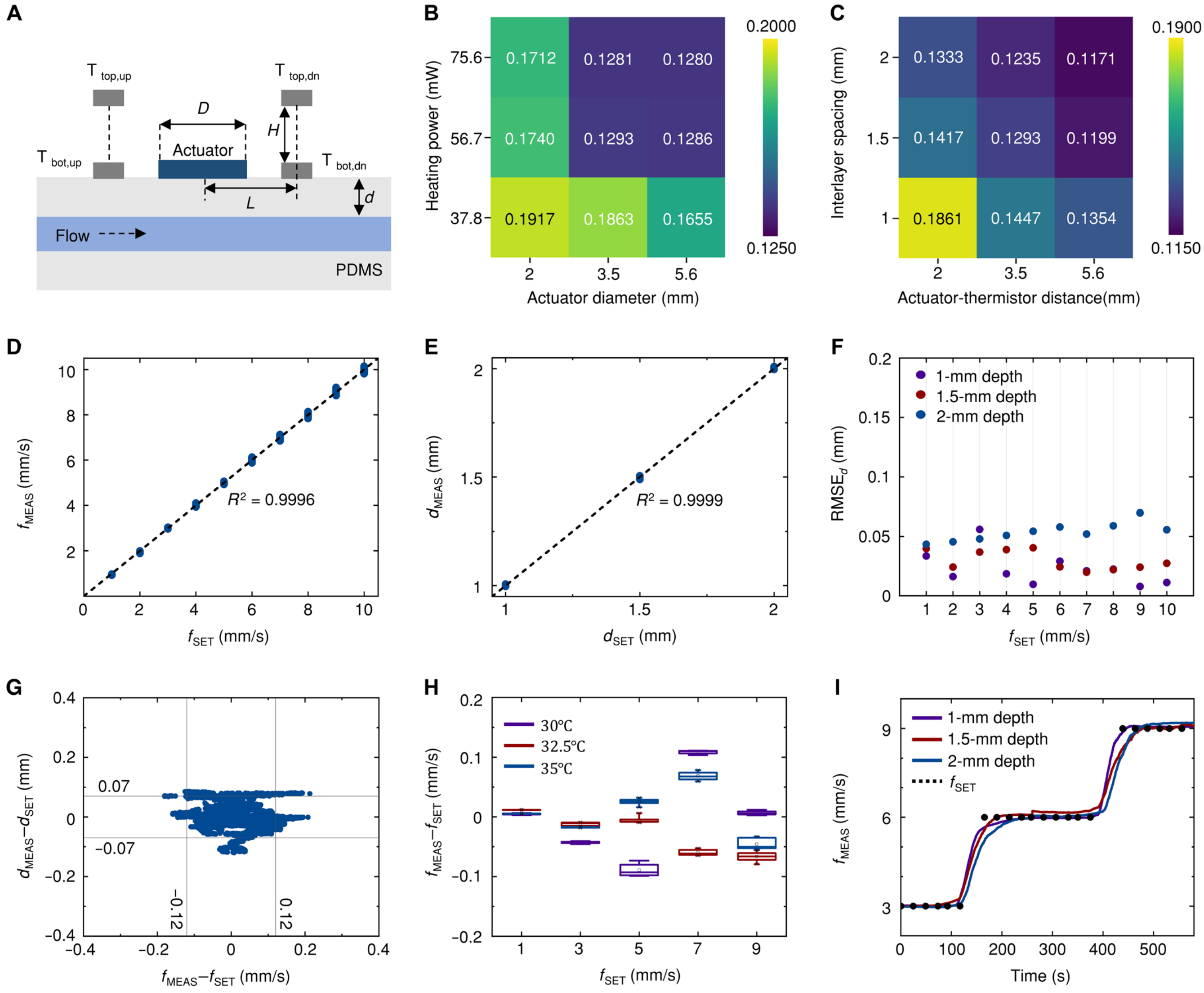

Fig. 3. System performance characterization and design optimization. (**A**) Schematic illustration of the thermal actuator geometry with multilayer thermistor configuration, defining design parameters: actuator diameter (D), heating power (P), actuator-thermistor distance (L), and interlayer spacing (H). (**B** and **C**) RMSE heatmaps for flow rate measurements across various design parameter combinations. (B) RMSE values for actuator diameter (2, 3.5, and 5.6 mm) and heating power (37.8, 56.7, and 75.6 mW) with $L = 3.5$ mm and $H = 1.5$ mm. (C) RMSE analysis for actuator-thermistor distance (2, 3.5, and 5.6 mm) and interlayer spacing (1, 1.5, and 2 mm) with $D = 3.5$ mm and $P = 56.7$ mW. (**D** and **E**) Linear correlation analysis using optimized parameters ($D = 3.5$ mm, $P = 56.7$ mW, $L = 3.5$ mm, $H = 1.5$ mm) on an expanded dataset of 9000 measurement points, demonstrating prediction accuracy for both flow rate [(D), $R^2 = 0.9996$] and vessel depth [(E), $R^2 = 0.9999$]. (**F**) Vessel depth RMSE across different flow rates, maintaining values below 0.07 mm for a 1- to 10-mm/s range. (**G**) Error distribution scatterplot showing measurement deviation with 95% of flow rate and vessel depth data points falling within ±0.12 mm/s and ±0.07 mm, respectively. (**H**) Flow rate measurement errors across ambient temperatures of 30°, 32.5°, and 35°C. (**I**) Flow rate measurements (solid lines) during step changes in flow velocity (3, 6, and 9 mm/s) across vessel depths of 1, 1.5, and 2 mm. Dotted line indicates set flow rates.

across flow rates of 1 to 10 mm/s. The scattering pattern in Fig. 3G demonstrates that f_{MEAS}–f_{SET} and d_{MEAS}–d_{SET} are tightly clustered around zero with minimal correlation, and 95% of data points fall within error margins of ±0.12 mm/s for flow rate and ±0.07 mm for vessel depth. FEA simulations investigated how variations in tissue and environmental parameters affect temperature distribution and heat transfer at the skin-vessel interface, thereby modifying the thermal gradients measured by the top and bottom thermistors. Figures S9 to S12 show minimum-maximum normalized sensor readings, $S_N = (T - T_{min})/(T_{max} - T_{min})$, across physiologically relevant ranges of skin thermal conductivities, blood characteristics (thermal conductivity and density), vessel properties (stiffness, wall thickness, and diameter), and ambient conditions (airflow). Skin thermal conductivity (0.32 to 0.42 W/m·K) and blood properties including thermal conductivity (0.5 to 0.6 W/m·K) and density (1025 to 1075 kg/m^3) induced maximum variations of $\Delta S_N = 0.066$, 0.008, and 0.013, respectively. Among vessel properties, wall stiffness (130 to 180 GPa) showed negligible effects ($\Delta S_N < 0.001$). In contrast, vessel diameter (2 to 3 mm) and wall thickness (0.4 to 0.6 mm) produced $\Delta S_N = 0.050$ and 0.099, respectively, at the baseline flow rate (1 mm/s), with variations becoming more pronounced at higher blood flow rates. Ambient airflow (0.05 to 0.2 m/s) induced variations of $\Delta S_N = 0.129$.

Figure 3H demonstrates device performance across various ambient temperatures. The system was trained using measurements obtained at 25°C and then tested at elevated temperatures of 30°, 32.5°, and 35°C. Testing was conducted at flow rates of 1, 3, 5, 7, and 9 mm/s, with 10 measurements collected for each flow rate condition. Results indicate that all measurement errors (f_{MEAS}–f_{SET}) remained within ±0.12mm/s across all tested temperature and flow rate combinations, demonstrating robust temperature stability. Figure 3I demonstrates dynamic measurement capabilities by measuring real-time flow rates during step changes in flow velocity (f_{SET} = 3, 6, and 9 mm/s) across vessels at different depths (d_{SET} = 1, 1.5, and 2 mm). The system accurately tracks flow dynamics, closely following the f_{SET} profile (dotted line). Notably, even as vessel depth increases, the system maintains comparable tracking accuracy, confirming robust performance across the full range of physiologically relevant vessel depths. Temporal response characterization (fig. S13) reveals depth-dependent dynamics, with rise times increasing with depth due to extended thermal diffusion paths through overlying tissue. While these experiments used abrupt flow transitions, physiological blood flow changes occur more gradually.

Human subject validation

The perfusion index (PI) is a widely accepted clinical parameter for capturing hemodynamic changes (*21*, *22*). While PI does not provide absolute blood flow values, it has been commonly used as a validation reference for blood flow velocity measurements in previous studies (*11*). In this study, PI served as the comparative benchmark for evaluating our blood flow rate measurements, with analysis focusing on correlations between blood flow rates and PI variations during controlled physiological situations. Figure 4A shows a photograph of our prototype placed on a subject's wrist with the sensor centered over a visible vein, alongside a reference PI device (Handheld Pulse Oximeter, Lepu Medical) attached to the index finger. The prototype performs temperature measurements at a sampling rate of 20 Hz and transmits these values to a UI. The UI stores the incoming data and averages the most recent 20 measurements (1-s window) from each thermistor to synchronize with the 1-Hz measurement rate of the PI device. These values are then fed into the deep learning algorithm, pretrained with in vitro experiments, for real-time flow rate prediction and correlation analysis with PI measurements.

Experimental validation was conducted on three healthy participants (see table S2 for details) using controlled physiological interventions to induce hemodynamic variations (Fig. 4B): respiratory modulation (participant 1, left wrist) involving 70-s breath-holding periods; external vascular compression (participant 2, right wrist) with two 60-s pressure applications using silicon wire (silicon hose, HSW) wrapped around the upper arm; and cycling exercise (participant 3, left wrist) with alternating low- and high-intensity cycling. All participants underwent a 10-min resting period before measurements to establish stable baseline conditions.

Results in Fig. 4 (C to E) demonstrate consistent correlations between blood flow rates and PI measurements across various physiological scenarios. During respiratory modulation experiments (Fig. 4C), both blood flow rate (blue line) and PI (black line) exhibited similar temporal patterns—decreasing synchronously during breath-holding periods, followed by coordinated recovery where both parameters increased after breath-holding ceased. The decrease in both parameters during breath-holding is consistent with the results of previously reported hemodynamic responses to respiratory perturbations (*23*, *24*). External vascular compression (Fig. 4D) produced simultaneous reductions in both parameters during pressure application to the upper arm and coordinated restoration upon pressure release. Cycling trials (Fig. 4E) showed coherent response patterns where both parameters slightly decreased during high-intensity cycling periods and then elevated the end of the exercise. These exercise-induced hemodynamic changes are consistent with prior studies, which have shown that high-intensity exercise leads to substantial metabolic and vascular adaptations, including increased muscle oxygen extraction and altered blood flow regulation (*25*–*27*). The scatterplots in Fig. 4F demonstrate consistent correlations between PI values and blood flow measurements across all three physiological scenarios: respiratory modulation (top), external vascular compression (middle), and cycling exercise (bottom). The correlation analyses support the observations of synchronized responses during the perturbation experiments. To further validate our approach, we conducted parallel experiments using laser Doppler flowmetry (LDF), which provides relative velocity measurements specifically optimized for superficial vessels (1- to 2-mm depth). Figure S14 shows correlations between TSM and LDF measurements across nine participants during vascular compression, confirming the device's capability to track relative flow changes. See table S3 and Materials and Methods for more details.

Thermal safety was evaluated using four different capacity batteries: 35, 90, 180 mAh, and 350 mAh. Battery temperature elevations after 10-min heating were <0.22°C (35 mAh), <0.11°C (90 mAh), <0.04°C (180 mAh), and <0.03°C (350 mAh) (for details, see fig. S15). All configurations maintained operation within safe thermal limits for skin-interfaced applications across the tested discharge rate range (0.049° to 0.49°C).

Continuous blood pressure monitoring

The blood flow sensing platform can integrate with various existing health care technologies to enhance diagnostic capabilities. As a demonstration of this integration potential, the platform was combined with PPG technology to improve continuous blood pressure measurement accuracy. Current wearable devices and conventional approaches using PPG and pulse transit time calculations fail to provide reliable, continuous blood pressure monitoring, offering only intermittent measurements lacking the precision required for clinical reliability in dynamic settings (*17*–*19*). This limitation proves particularly problematic for detecting acute hypertensive or hypotensive events such as shock (*28*, *29*), where moment-to-moment blood pressure changes serve as critical diagnostic indicators (*14*, *30*). The integrated approach combines thermal blood flow measurements with PPG signals to improve measurement accuracy while providing continuous diastolic/systolic blood pressure (DP/SP) monitoring capabilities.

Figure 5A illustrates the experimental setup: TSM positioned over a wrist vein, a PPG sensor fabricated on a fPCB (see Materials and Methods and fig. S16 for more details) on the middle finger, and a clinical-grade blood pressure monitor (Finapres NOVA, Finapres Medical Systems) on the index finger for reference blood pressure measurements (DP_{REF} and SP_{REF}). Participants remained seated throughout the measurement period to ensure stable reference measurements and minimize motion artifacts. See Materials and Methods for detailed specifications and measurement principles. Figure 5B shows the neural net architectures to process the collected signals. The parallel pathways independently process TSM data through long short-term memory (LSTM) layers and PPG signals through convolutional

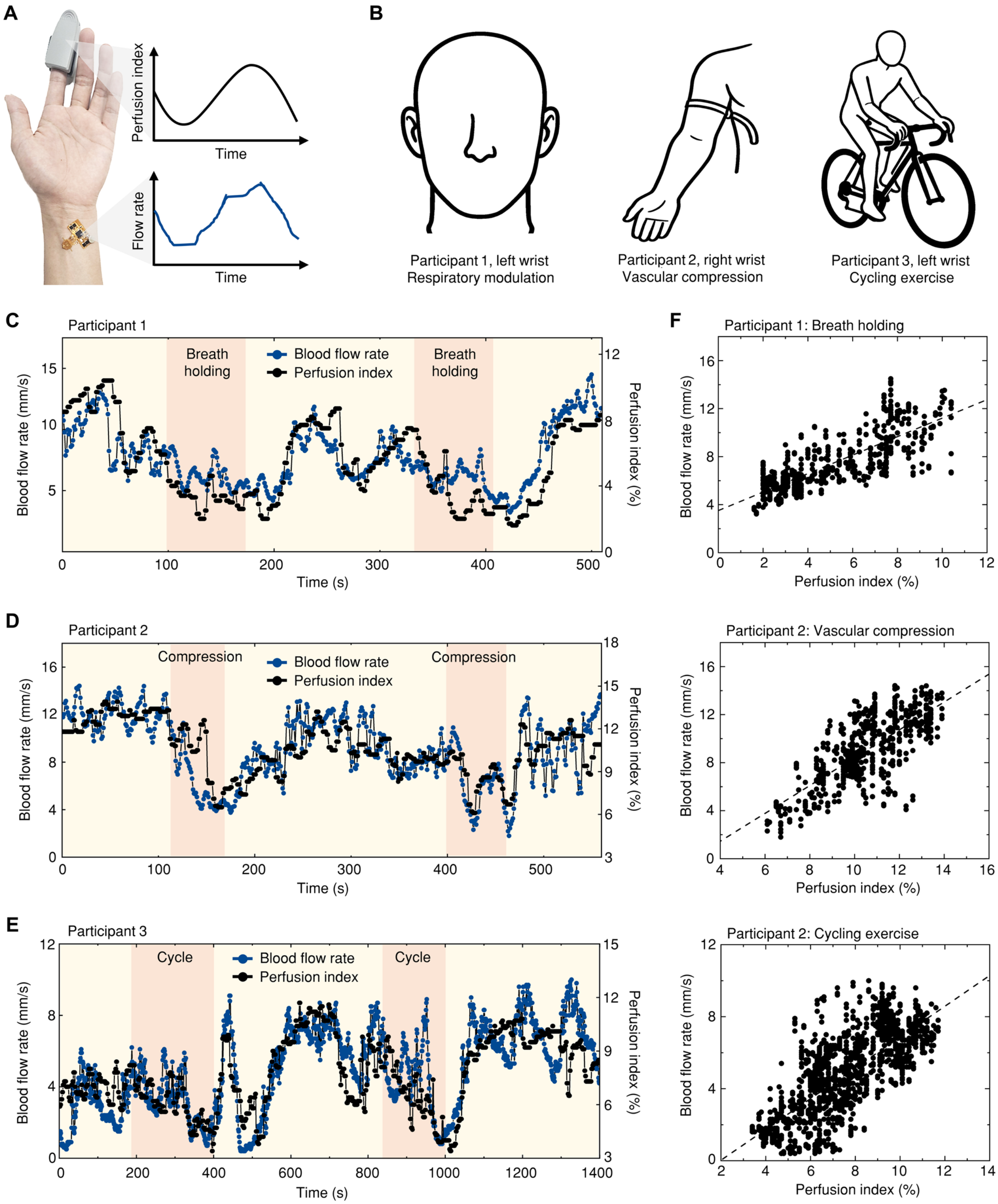

Fig. 4. Human subject validation through controlled physiological interventions. (**A**) Optical image of the blood flow sensing device positioned on a subject's wrist over a visible vein with a reference PI device attached to the index finger. (**B**) Experimental setup for three physiological interventions: respiratory modulation (participant 1), external vascular compression (participant 2), and cycling exercise (participant 3). (**C** to **E**) Time-course measurements of blood flow rate (blue lines) and PI (black lines) during breath-holding (C), vascular compression (D), and cycling exercise (E). (**F**) Correlation scatterplots between PI values and blood flow measurements of all three physiological scenarios: respiratory modulation (top), external vascular compression (middle), and cycling exercise (bottom).

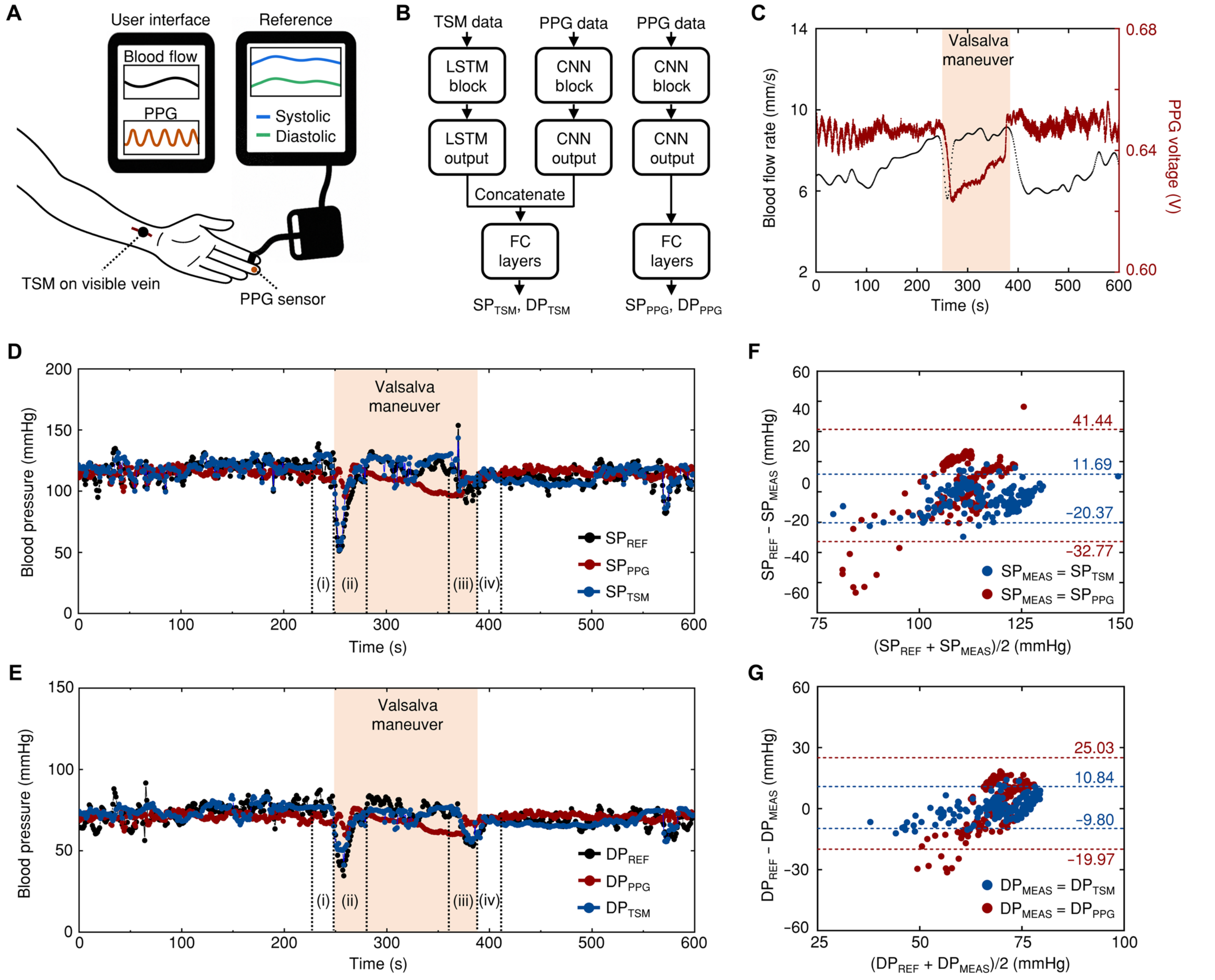

Fig. 5. Continuous blood pressure monitoring through thermal sensing and PPG integration. (**A**) Experimental setup illustration showing the TSM positioned over a wrist vein, PPG sensor on the middle finger, and clinical-grade blood pressure monitor (Finapres NOVA) on the index finger for reference measurements. (**B**) Neural network architecture for blood pressure prediction featuring parallel pathways that independently process TSM and PPG data through LSTM and CNN components, respectively. After concatenation, fully connected (FC) layers generate real-time blood pressure estimates (SP_{TSM} and DP_{TSM}). (**C**) Simultaneous measurements of blood flow rate (black line) and PPG sensor (red line) over 600 s. The participant performed a Valsalva maneuver for 140 s (highlighted regions) to induce rapid blood pressure changes. (**D** and **E**) Blood pressure predictions comparing reference values (black lines; SP_{REF} and DP_{REF}) with PPG-only predictions (red; SP_{PPG} and DP_{PPG}) and integrated TSM-PPG predictions (blue; SP_{TSM} and DP_{TSM}) for systolic (D) and diastolic (E) pressures. (**F** and **G**) Bland-Altman plot for systolic (F) and diastolic (G) measurement (SP_{MEAS} and DP_{MEAS}) agreement during Valsalva maneuver and transition phases (10 s before, 140 s during, and 30 s after). Data from the participant in (D) and (E) (one subject, 180 samples).

neural network (CNN) components (*31–33*). After concatenating these outputs, fully connected layers generate blood pressure estimates. See text S2 for details in the complete architectural specifications. Performance comparison between PPG-only (DP_{PPG} and SP_{PPG}) and integrated TSM-PPG (DP_{TSM} and SP_{TSM}) approaches assessed the contribution of thermal flow sensing.

Figure 5C shows simultaneous measurements of blood flow rate (black line) and PPG sensor output (red line) captured over a 600-s measurement period. To induce rapid blood pressure changes and evaluate the system's ability to track dynamic cardiovascular responses, the participant performed a Valsalva maneuver (*34*) for 140 s during the measurement session (highlighted regions). This intervention creates distinct patterns in both signals—gradual decline in blood flow rate accompanied by changes in PPG amplitude. Blood pressure predictions derived from these signals are compared with reference measurements in Fig. 5 (D and E) for SP and DP, respectively. Figure 5 (D and E) displays the reference values (black lines; SP_{REF} and DP_{REF}) alongside the corresponding predictions from PPG-only (red; SP_{PPG} and DP_{PPG}) and integrated TSM-PPG approaches (blue; SP_{TSM} and DP_{TSM}). Figure S17 presents both SP and DP in a single panel. During the Valsalva maneuver (highlighted regions), blood pressure undergoes a characteristic four-phase response: (i) initial elevation due

to increased intrathoracic pressure, (ii) compensatory decrease as venous return diminishes, (iii) transient drop upon pressure release, and (iv) overshoot before returning to baseline (*35*). The PPG-only method exhibits notable deviations from reference measurements during these rapid phase transitions, particularly in phases ii and iii when compensatory mechanisms activate and upon pressure release, causing substantial hemodynamic fluctuations. In contrast, the integrated approach demonstrates substantially improved tracking accuracy throughout all four phases of the Valsalva response, with predictions closely following reference values even during these challenging hemodynamic transitions (for details, see fig. S18). Phase-specific analysis (fig. S19) reveals distinct TSM integration contributions. During baseline conditions (pre- and post-Valsalva), TSM integration reduces measurement error by 36.2 and 9.54% for SP and DP, respectively, compared to PPG-only methods. During dynamic phases, phases i to iv show error reductions of 12.2/55.8, 59.7/60.7, 63.9/51.7, and 25.2/72.6% for SP and DP, respectively, with the largest benefit during phases ii and iii when blood pressure undergoes the most dynamic changes.

Bland-Altman analysis in Fig. 5 (F and G) further quantifies this complementary relationship during the complete hemodynamically challenging period—the Valsalva maneuver and its immediate transition phases (10 s before, 140 s during, and 30 s after the maneuver). For SP (Fig. 5F), the PPG-only approach shows 95% limits of agreement (LoA) ranging from −32.77 to +41.44 mmHg, while the integrated approach achieves narrowed limits of −20.37 to +11.69 mmHg. For DP (Fig. 5G), the PPG-only method shows limits from −19.97 to +25.03 mmHg, whereas the integrated approach achieves −9.80 to +10.84 mmHg. The substantially narrower LoA confirms improved precision with the integrated method even during rapid hemodynamic transitions.

DISCUSSION

This work presents a skin-interfaced electronic platform for real-time, wireless monitoring of blood flow rate and vessel depth using multilayer thermal sensing integrated with deep learning techniques. Unlike conventional single-layer sensing approaches that fail to account for vessel-depth variations, the platform successfully distinguishes between flow rate and depth variables through vertically spaced sensing layers equipped with thermistors and a thermal actuator. The deep learning framework analyzes the multilayer thermal patterns to simultaneously extract both parameters with high precision across the physiologically relevant ranges.

Our platform can interface with various health care technologies, demonstrated through PPG integration for improved blood pressure measurement accuracy during rapid hemodynamic fluctuations. This integration addresses continuous, wearable blood pressure monitoring by combining the accuracy of clinical systems with the mobility of portable devices while eliminating cuff-based constraints. The approach has the potential to enable monitoring across diverse patient populations, including those where conventional cuff placement presents challenges. While our TSM-PPG integration demonstrated improved tracking during Valsalva maneuvers, broader testing across diverse hemodynamic conditions is required to establish clinical utility. Comprehensive validation requires clinical studies in patient populations with natural blood pressure variations from pathological states, pharmacological interventions, and circadian rhythms to assess performance across clinically relevant scenarios. Future investigations could explore personalized calibration strategies, where individual fine-tuning of the deep learning model may further enhance measurement precision for patient-specific hemodynamic monitoring and personalized cardiovascular care. Additional integration possibilities include combination with pulse wave velocity systems to deliver comprehensive cardiovascular evaluations or deployment in intensive care scenarios for continuous perfusion monitoring. This capability supports ongoing cardiovascular risk assessment, advancing personalized and proactive medical care for both health care providers and patients.

The technology offers additional opportunities in the management and diagnosis of cardiovascular and circulatory conditions. Applications include automated alerts for sudden hemodynamic events such as shock or hypotensive episodes, continuous surveillance for peripheral vascular disorders, and ongoing skin graft perfusion monitoring to enable early identification of vascular complications and therapeutic intervention assessment. Future optimization, incorporating established scaling laws for thermal conductivity, heat capacity, and vascular geometry, can enhance device performance across these medical applications. Direct experimental validation with actual blood, rather than water-based benchtop models, could further improve model accuracy by better representing physiological heat transfer characteristics. Advanced battery management systems incorporating battery voltage/temperature monitoring and adaptive discharge rate regulation represent important future research directions for enhancing device longevity and safety.

While our system demonstrates blood pressure monitoring under controlled conditions, extending validation to dynamic physiological conditions with appropriate artifact suppression strategies represents an important next step for practical ambulatory monitoring. In addition, future clinical translation will require extensive validation across diverse populations including variations in skin phototypes, body mass index ranges, age groups, and relevant cardiovascular pathologies to ensure broad clinical applicability.

MATERIALS AND METHODS

Finite element analysis

FEA using COMSOL Multiphysics provided thermal transport characteristics for multilayer sensor design optimization. The analysis used a three-dimensional *Heat Transfer in Solids and Fluids* physics module to simulate the thermal interactions between the actuator, sensing layers, and flowing medium. The computational model consisted of a poly(dimethylsiloxane) (PDMS) structure containing an embedded cylindrical channel with flowing water to simulate blood vessels, a copper thermal actuator on the top surface, and thermistors at upstream and downstream positions in both top and bottom sensing layers. The interlayer medium between the two sensing layers was air to reflect the actual device architecture, with acrylic supports maintaining physical separation. Boundary conditions included thermally insulated lateral surfaces of the PDMS structure, with the bottom surface maintained at 25°C and the top surface featuring convective heat flux with a heat transfer coefficient of 15 W/m^{2}K. All components were initialized at 25°C. Material properties were as follows: PDMS (heat capacity: 1460 J/kg·K, thermal conductivity: 0.16 W/m·K), water representing blood flow (4184 J/kg·K, 0.6 W/m·K), and copper actuator (385 J/kg·K, 400 W/m·K). Flow conditions used inlet velocity boundary conditions at the channel entrance with water entering at 25°C, while the thermal actuator used a uniform boundary heat source across the entire copper surface. Figure S3 provides detailed FEA model information.

Device fabrication
AutoCAD 2022 (Autodesk) provided initial circuit layout design for the fPCB, with Altium Designer 17 (Altium Limited) converting designs to Gerber files for manufacturing preparation. Initial prototype studies served as the basis for final designs provided to an ISO-9001 compliant vendor (PCBWay) for manufacturing. The fPCB featured a thickness of 0.1 mm with immersion gold (ENIG, 1 U") surface finish for reliable electrical connections and corrosion resistance. The electronic system incorporated a BLE SoC (nRF52832, Nordic Semiconductor) for wireless communication and signal processing, along with amplifiers (INA333, Texas Instruments), antenna (2450AT18A100, Johanson Technology), and low-dropout regulator (ISL9016IRUNCZ, Renesas Electronics Corporation) for power management. Thermal gradient sensors include 10-kΩ thermistors (NCP03XH103E05RL, Murata Electronics) as temperature sensors and resistors (R1005G24-R0F1-16W50V, WALSIN Technology) as thermal actuators. Component assembly was performed using precision soldering techniques with a microscope (26800B-371, Aven Tools) for visual guidance, soldering paste (TS391LT, Chip Quik) for surface-mount device attachment, and a heat gun (858D, Dinkke) for reflow soldering. BLE SoC firmware programming used SEGGER Embedded Studio, with code deployment via SWDIO and SWDCLK pin connections between the fPCB-mounted BLE SoC and an nRF52DK development kit (Nordic Semiconductor) serving as the programming interface. The wireless system operates with a current consumption of 17.2 mA during active thermal sensing and BLE transmission, achieved through three parallel-connected GPIO pins (5.73 mA each) to operate within individual pin current limits and <0.1 mA during standby mode. BLE communication testing showed reliable data transmission with no packet loss at distances below 25 m (1, 5, 10, 15, 20, and 25 m) and 5.03% packet loss at 30 m in typical indoor environments. To achieve maximum miniaturization, the current prototype uses a compact 5.5-mAh coin cell battery (6.8 mm by 2.1 mm), supporting various monitoring scenarios: 1.8 days for once-daily measurements (10-min sessions), 7.6 hours for every-4-hour monitoring, 3.8 hours for every-2-hour monitoring, or 1.9 hours for hourly monitoring. The modular platform design readily accommodates larger capacity batteries for extended clinical applications. Acrylic support fabrication involved designing the support structures using AutoCAD 2022, followed by cutting acrylic sheets using a laser cutter (VLS 3.50, Universal Laser Systems) to maintain separation between the top and bottom sensing layers, with detailed specifications provided in fig. S4. This fabrication approach enabled precise control over the multilayer sensor geometry while maintaining the flexibility required for skin-conformal applications.

IR thermal imaging
A FLIR a400 camera (FLIR Systems) equipped with a 17-mm lens visualized heat distribution patterns generated by the thermal actuator. LIR Research Studio 3.2.3 software acquired and recorded images for real-time thermal pattern analysis and validation of the multilayer sensing approach.

Benchtop validation
Benchtop validation used custom-fabricated vessel phantoms consisting of PDMS blocks (Sylgard 184, 10:1 base to curing agent ratio) with embedded silicon tubing (silicon hose, HSW) to simulate blood vessels. The vessel phantoms featured 1 cm–by–8 cm–by–9 cm PDMS blocks containing 2.5-mm-diameter silicon tubing (silicon hose, HSW) positioned at controlled depths. AutoCAD enabled mold design for the phantoms, with subsequent Stereolithography (.stl) file conversion and fabrication using a three-dimensional printer (3DWOX1, Sindoh Co.) with 5-mm wall thickness. PDMS casting into the molds and subsequent hot plate curing at 150°C for 1 hour finalized the vessel phantom fabrication, with detailed specifications shown in fig. S20.

A constant temperature humidity chamber (HPP410eco) maintained controlled environmental conditions for all benchtop experiments, with device power supplied by a dc power supply (DP832A, Rigol). Initial phantom fabrication at 1-mm vessel depth, followed by addition of 0.5-mm PDMS layers, created 1.5- and 2-mm depth configurations for vessel depth variations. Preparation of the 0.5-mm PDMS layers involved pouring PDMS onto slide glass substrates (76 mm by 52 mm, 1.5-mm thickness, Matsunami) and spin coating at 200 rpm for 20 s using a spin coater (SF-100ND, Rhabdos), followed by curing at 100°C for 10 min. A syringe pump (NE-1000, New Era) operating in withdrawal mode generated flow conditions spanning 1 to 10 mm/s, drawing temperature-controlled water from a constant temperature (25°C) water bath (Digital Water Bath, LABTron) maintained at chamber ambient temperature, ensuring precise thermal equilibrium between the flowing medium and experimental environment. The wireless systems transmitted measured data to an external smartphone at a 20-Hz sampling rate via BLE communication, with an Android application developed using Android Studio Bumblebee handling data acquisition by storing real-time measurements as text files. An additional Android application performed real-time inference for flow rate and vessel depth determination, continuously reading the most recent data from the stored text files and processing them through the deep learning inference layers. Figure S6 provides detailed information about the Android applications.

Deep learning implementation
A server equipped with Intel Xeon W-3300 processors and dual NVIDIA A6000 D6 48GB PCIe graphics processing units (GPUs) performed deep learning model training and inference. Visual Studio Code (Microsoft) managed model development and server connectivity, using PyTorch framework with Compute Unified Device Architecture (CUDA) 12.6 for GPU acceleration. Texts S1 and S2 provide detailed specifications of the neural network architecture and training parameters.

LDF validation
LDF measurements were performed using the PeriFlux System 5000 (PERIMED) to provide additional validation of blood flow measurements. The TSM device was positioned on the subject's wrist over a visible vein, with the LDF probe placed adjacent to the device to measure the same vascular region. Each validation session consisted of 250-s measurement periods, during which vascular compression was applied for 60 s via silicon tubing wrapped around the upper arm to induce controlled hemodynamic changes. Participants self-reported their skin type according to the Fitzpatrick scale after being shown reference images and descriptions.

PPG sensor configuration
The PPG sensor module uses an optical sensor (MAXM86161, Analog Devices) for blood volume pulse detection. The sensor circuit includes peripheral components consisting of 10- and 1-μF capacitors for signal conditioning and power supply stabilization. The sensor communicates with the BLE SoC through Inter-Integrated Circuit protocol, transmitting measurement data at a sampling rate of 128 Hz for real-time signal acquisition.

Clinical-grade blood pressure monitoring

Reference blood pressure measurements used a Finapres NOVA Noninvasive Hemodynamic Monitor (Finapres Medical Systems), a cuff-based continuous blood pressure monitoring system that has received U.S. Food and Drug Administration (FDA) 510 (k) premarket notification approval. The device is classified as a class II medical device under 21 CFR 870.1130 (noninvasive blood pressure measurement system) regulations, with FDA clearance numbers K173916, K160967, and K141460 demonstrating "substantially equivalent" determination to predicate devices. The Finapres NOVA provides beat-to-beat arterial blood pressure measurements through volume-clamp methodology, delivering continuous systolic and diastolic pressure readings with clinical-grade accuracy. The system was positioned on the index finger of participants during all blood pressure validation experiments. Automated calibration procedures were performed according to the manufacturer's specifications before each measurement session to ensure measurement accuracy and reliability throughout the experimental protocol.

Human participant studies

The Korea Advanced Institute of Science and Technology (KAIST) Institutional Review Board for Human Subjects Research approved human subject experiments (Institutional Review Board approval number: KH2023-149). All participants provided informed consent before experimentation and received comprehensive explanations of the experimental procedures, which posed no safety risks. Healthy adult volunteers aged 23 to 27 years (both male and female) participated in controlled physiological interventions including respiratory modulation, wrist compression, intense cycling, and Valsalva maneuver following detailed preexperimental instruction.

During experiments, the wireless blood flow sensing platform continuously recorded data using the same Android application described previously, while reference measurements occurred simultaneously using a PI device (Handheld Pulse Oximeter, Lepu Medical) and continuous blood pressure monitor (Finapres NOVA, Finapres Medical Systems) depending on the specific experimental protocol. Oximeter Data Manager computer software recorded data from the PI device, while the Finapres NOVA stored data internally for subsequent extraction via universal serial bus (USB) interface.

Supplementary Materials

The PDF file includes:
Text S1 and S2
Figs. S1 to S20
Tables S1 to S3
Legend for movie S1
References

Other Supplementary Material for this manuscript includes the following:
Movie S1

REFERENCES

1. K. Kisler, A. R. Nelson, A. Montagne, B. V. Zlokovic, Cerebral blood flow regulation and neurovascular dysfunction in Alzheimer disease. *Nat. Rev. Neurosci.* **18**, 419–434 (2017).
2. G. Eelen, P. De Zeeuw, M. Simons, P. Carmeliet, Endothelial cell metabolism in normal and diseased vasculature. *Circ. Res.* **116**, 1231–1244 (2015).
3. B. V. Zlokovic, R. F. Gottesman, K. E. Bernstein, S. Seshadri, A. McKee, H. Snyder, S. M. Greenberg, K. Yaffe, C. B. Schaffer, C. Yuan, T. M. Hughes, M. J. Daemen, J. D. Williamson, H. M. González, J. Schneider, C. L. Wellington, Z. S. Katusic, L. Stoeckel, J. I. Koenig, R. A. Corriveau, L. Fine, Z. S. Galis, J. Reis, J. D. Wright, J. Chen, Vascular contributions to cognitive impairment and dementia (VCID): A report from the 2018 National Heart, Lung, and Blood Institute and National Institute of Neurological Disorders and Stroke Workshop. *Alzheimers Dement.* **16**, 1714–1733 (2020).
4. "A discussion on the regulation of blood flow and pressure" in *Advances in Experimental Medicine and Biology* (Springer New York, 2016), pp. 129–135; http://link.springer.com/10.1007/978-1-4939-3023-4_16.
5. L. Sonnenschein, T. Etyang, Blood flow and health. *AJBSR* **22**, 374–392 (2024).
6. R. M. Bober, R. V. Milani, S. M. Kachur, D. P. Morin, Assessment of resting myocardial blood flow in regions of known transmural scar to confirm accuracy and precision of 3D cardiac positron emission tomography. *EJNMMI Res.* **13**, 87 (2023).
7. B. Kerimkhan, A. Nedzved, A. Zhumadillayeva, K. Dyussekeyev, G. Uskenbayeva, B. Sultanova, L. Rzayeva, Automation of flow analysis in scleral vessels based on descriptive-associative algorithms. *Sci. Rep.* **13**, 4650 (2023).
8. M. K. Razavi, D. P. T. Flanigan, S. M. White, T. B. Rice, A real-time blood flow measurement device for patients with peripheral artery disease. *J. Vasc. Interv. Radiol.* **32**, 453–458 (2021).
9. P. Blanco, Volumetric blood flow measurement using Doppler ultrasound: Concerns about the technique. *J. Ultrasound* **18**, 201–204 (2015).
10. A. Oglat, M. Matjafri, N. Suardi, M. Oqlat, M. Abdelrahman, A. Oqlat, A review of medical doppler ultrasonography of blood flow in general and especially in common carotid artery. *J. Med. Ultrasound* **26**, 3 (2018).
11. R. C. Webb, Y. Ma, S. Krishnan, Y. Li, S. Yoon, X. Guo, X. Feng, Y. Shi, M. Seidel, N. H. Cho, J. Kurniawan, J. Ahad, N. Sheth, J. Kim, J. G. Taylor Vi, T. Darlington, K. Chang, W. Huang, J. Ayers, A. Gruebele, R. M. Pielak, M. J. Slepian, Y. Huang, A. M. Gorbach, J. A. Rogers, Epidermal devices for noninvasive, precise, and continuous mapping of macrovascular and microvascular blood flow. *Sci. Adv.* **1**, e1500701 (2015).
12. K. Kwon, J. U. Kim, Y. Deng, S. R. Krishnan, J. Choi, H. Jang, K. Lee, C.-J. Su, I. Yoo, Y. Wu, L. Lipschultz, J.-H. Kim, T. S. Chung, D. Wu, Y. Park, T. Kim, R. Ghaffari, S. Lee, Y. Huang, J. A. Rogers, An on-skin platform for wireless monitoring of flow rate, cumulative loss and temperature of sweat in real time. *Nat. Electron.* **4**, 302–312 (2021).
13. Y. Deng, H. M. Arafa, T. Yang, H. Albadawi, R. J. Fowl, Z. Zhang, V. Kandula, A. Ramesh, C. Correia, Y. Huang, R. Oklu, J. A. Rogers, A. S. Carlini, A soft thermal sensor for the continuous assessment of flow in vascular access. *Nat. Commun.* **16**, 38 (2025).
14. J. Fortin, D. E. Rogge, C. Fellner, D. Flotzinger, J. Grond, K. Lerche, B. Saugel, A novel art of continuous noninvasive blood pressure measurement. *Nat. Commun.* **12**, 1387 (2021).
15. E. M. Lee, When and how to use ambulatory blood pressure monitoring and home blood pressure monitoring for managing hypertension. *Clin. Hypertens.* **30**, 10 (2024).
16. A. Dadlani, K. Madan, J. P. S. Sawhney, Ambulatory blood pressure monitoring in clinical practice. *Indian Heart J.* **71**, 91–97 (2019).
17. Y. Degiorgis, M. Proença, Y. Ghamri, G. Hofmann, M. Lemay, P. Schoettker, Photoplethysmography-based blood pressure monitoring could improve patient outcome during anesthesia induction. *J. Pers. Med.* **12**, 1571 (2022).
18. D. Konstantinidis, P. Iliakis, F. Tatakis, K. Thomopoulos, K. Dimitriadis, D. Tousoulis, K. Tsioufis, Wearable blood pressure measurement devices and new approaches in hypertension management: The digital era. *J. Hum. Hypertens.* **36**, 945–951 (2022).
19. M. Elgendi, F. Haugg, R. R. Fletcher, J. Allen, H. Shin, A. Alian, C. Menon, Recommendations for evaluating photoplethysmography-based algorithms for blood pressure assessment. *Commun. Med.* **4**, 140 (2024).
20. C. Tantithamthavorn, S. McIntosh, A. E. Hassan, K. Matsumoto, An empirical comparison of model validation techniques for defect prediction models. *IIEEE Trans. Software Eng.* **43**, 1–18 (2017).
21. M. M. Elshal, A. M. Hasanin, M. Mostafa, R. M. Gamal, Plethysmographic peripheral perfusion index: Could it be a new vital sign? *Front. Med.* **8**, 651909 (2021).
22. K. Gupta, B. Rastogi, P. K. Gupta, M. K. Bansal, P. Kalra, S. Kaur, Perfusion index to assess hemodynamic changes and analgesia with premedication of fentanyl versus nalbuphine during general anesthesia. *AAN* **4**, 255–259 (2019).
23. A. Grunovas, E. Trinkunas, A. Buliuolis, E. Venskaityte, J. Poderys, K. Poderiene, Cardiovascular response to breath-holding explained by changes of the indices and their dynamic interactions. *Biol. Syst. Open Access* **05**, 1000152 (2015).
24. I. Taneja, M. S. Medow, D. A. Clarke, A. J. Ocon, J. M. Stewart, Postural change alters autonomic responses to breath-holding. *Clin. Auton. Res.* **20**, 65–72 (2010).
25. M. E. Moir, A. T. Corkery, K. B. Miller, A. G. Pearson, N. A. Loggie, A. A. Apfelbeck, A. J. Howery, J. N. Barnes, The independent and combined effects of aerobic exercise intensity and dose differentially increase post-exercise cerebral shear stress and blood flow. *Exp. Physiol.* **109**, 1796–1805 (2024).
26. S. K. Nyberg, O. K. Berg, J. Helgerud, E. Wang, Blood flow regulation and oxygen uptake during high-intensity forearm exercise. *J. Appl. Physiol.* **122**, 907–917 (2017).
27. M. T. Chua, A. Sim, S. F. Burns, Acute and chronic effects of blood flow restricted high-intensity interval training: A systematic review. *Sports Med. Open* **8**, 122 (2022).
28. A. Bergholz, G. Greiwe, K. Kouz, B. Saugel, Continuous blood pressure monitoring in patients having surgery: A narrative review. *Medicina* **59**, 1299 (2023).
29. R. Mukkamala, J.-O. Hahn, O. T. Inan, L. K. Mestha, C.-S. Kim, H. Toreyin, S. Kyal, Toward ubiquitous blood pressure monitoring via pulse transit time: Theory and practice. *IEEE Trans. Biomed. Eng.* **62**, 1879–1901 (2015).

30. M. Komolova, M. A. Adams, Moment-to-moment characteristics of the relationship between arterial pressure and renal interstitial hydrostatic pressure. *Hypertension* **56**, 650–657 (2010).
31. J. Perdereau, T. Chamoux, E. Gayat, A. Le Gall, F. Vallée, J. Cartailler, J. Joachim, Blood pressure estimation using explainable deep-learning models based on photoplethysmography. *Anesth. Analg.* **140**, 119–128 (2025).
32. D. U. Jeong, K. M. Lim, Combined deep CNN–LSTM network-based multitasking learning architecture for noninvasive continuous blood pressure estimation using difference in ECG-PPG features. *Sci. Rep.* **11**, 13539 (2021).
33. B. Kamanditya, Y. N. Fuadah, N. Q. Mahardika T., K. M. Lim, Continuous blood pressure prediction system using Conv-LSTM network on hybrid latent features of photoplethysmogram (PPG) and electrocardiogram (ECG) signals. *Sci. Rep.* **14**, 16450 (2024).
34. Y. Park, H. Luan, K. Kwon, T. S. Chung, S. Oh, J.-Y. Yoo, G. Chung, J. Kim, S. Kim, S. S. Kwak, J. Choi, H.-P. Phan, S. Yoo, H. Jeong, J. Shin, S. M. Won, H.-J. Yoon, Y. H. Jung, J. A. Rogers, Soft, full Wheatstone bridge 3D pressure sensors for cardiovascular monitoring. *NPJ Flex. Electron.* **8**, 6 (2024).
35. D. S. Goldstein, W. P. Cheshire, Beat-to-beat blood pressure and heart rate responses to the Valsalva maneuver. *Clin. Auton. Res.* **27**, 361–367 (2017).
36. F. Pedregosa, G. Varoquaux, N. Org, A. Gramfort, V. Michel, B. Thirion, O. Grisel, M. Blondel, P. Prettenhofer, R. Weiss, V. Dubourg, J. Vanderplas, A. Passos, D. Cournapeau, M. Brucher, M. Perrot, E. Duchesnay, Scikit-learn: Machine learning in python. *J. Mach. Learn. Res.* **18**, 2825–2830 (2011).

Acknowledgments

Funding: This work was supported by the National Research Foundation of Korea (NRF) grants funded by the Korea government (MSIP; Ministry of Science, ICT and Future Planning; no. 2022R1C1C1010555 and no. 2020R1A5A8018367) (Y.S. and K.K.), BK21 FOUR program through the National Research Foundation of Korea (NRF) under the Ministry of Education (Y.S. and K.K.), and Institute of Information and Communications Technology Planning and Evaluation (IITP) under the Graduate School of Artificial Intelligence Semiconductor (IITP-2024-RS-2023-00256472) grant funded by the Korea government (MSIT) (Y.S. and K.K.). Additional support was provided by the Samsung Advanced Institute of Technology (SAIT). **Author contributions:** Conceptualization: Y.S. and K.K. Methodology: Y.S. and K.K. Software: Y.S., Y.P., and K.K. Validation: Y.S. and K.K. Formal analysis: Y.S. and K.K. Investigation: Y.S. and Y.P. Resources: Y.S. and K.K. Data curation: Y.S. Writing—original draft: Y.S. and K.K. Writing—review and editing: Y.S. and K.K. Visualization: Y.S. and K.K. Supervision: Y.S. and K.K. Project administration: Y.S. and K.K. Funding acquisition: K.K. **Competing interests:** The authors declare that they have no competing interests. **Data and materials availability:** All data and code needed to evaluate and reproduce the results in the paper are present in the paper and/or the Supplementary Materials. This study did not generate new materials.

Submitted 25 July 2025
Accepted 5 January 2026
Published 6 February 2026
10.1126/sciadv.aea8902
