# Supplementary material for: Deep learning–integrated multilayer thermal gradient sensing platform for real-time blood flow monitoring
Source: Sci Adv. 2026 Feb 6;12(6):eaea8902. doi: 10.1126/sciadv.aea8902 (PMC12880533; doi:10.1126/sciadv.aea8902)
Supplement: Supplementary file 2 — Text S1 and S2 Figs. S1 to S20 Tables S1 to S3 Legend for movie S1 References [file sciadv.aea8902_sm.pdf]

Science Advances
AAAS

Supplementary Materials for

Deep learning–integrated multilayer thermal gradient sensing platform for real-time blood flow monitoring

Youngmin Sim *et al.*

Corresponding author: Kyeongha Kwon, kyeongha@kaist.ac.kr

Sci. Adv. **12**, eaea8902 (2026)
DOI: 10.1126/sciadv.aea8902

The PDF file includes:

Text S1 and S2
Figs. S1 to S20
Tables S1 to S3
Legend for movie S1
References

Other Supplementary Material for this manuscript includes the following:

Movie S1

Supplementary Text 1. Neural network architecture for flow rate and vessel depth monitoring

Training data generation and preprocessing

Input features comprised six thermal sensor readings: upstream and downstream signals from both sensing layers and reference thermistor readings. Vessel depth values were normalized from the original range (1.0 to 2.0 mm) to a compressed scale (0.1 to 0.2) to improve numerical stability during training. All input features underwent standardization using MinMaxScaler (*36*).

Network architecture and implementation

The neural network consists of four fully connected (linear) layers without hidden layers between the main processing stages. The first layer maps six input features to 128 nodes, followed by ReLU activation, and batch normalization. Two intermediate layers maintain 128 nodes each with ReLU activation and dropout (10 %) applied after the second layer. The final output layer reduces to two nodes corresponding to flow rate and vessel depth predictions without activation functions, enabling direct regression outputs. The architecture employs no data augmentation techniques, focusing on robust feature extraction from multi-layer thermal signatures.

Cross-validation methodology

Model robustness was evaluated using 5-fold cross-validation. The training process incorporated early stopping (patience = 50 epochs) and adaptive learning rate scheduling (Reduced by 0.5, patience = 20 epochs) to prevent overfitting and optimize convergence. Training was conducted using Adam optimizer with an initial learning rate of 0.001.

Supplementary Text 2. Neural network architecture specifications for integrated blood pressure monitoring

Data preprocessing and signal conditioning

PPG signals are initially acquired at 128 Hz using the Maxm86161 sensor and subsequently downsampled to 20 Hz through interpolation to match the temporal resolution of the TSM data. The dataset consists of 1200 seconds of continuous measurements, with the first 600 seconds used for training and the subsequent 600 seconds reserved for testing. Temperature sensor data normalization employs training set statistics with per-channel mean and standard deviation:

$$TSM_{test,\,normalized} = \frac{\left(TSM_{test} - mean_{TSM,\,train}\right)}{std_{TSM,\,train}}$$

CNN pathway architecture specifications

The PPG processing pathway employs a 1D convolutional architecture with two Conv1D layers (1 → 16 → 32 channels, kernel = 3) each followed by ReLU activation and MaxPool1D (kernel = 2), reducing the temporal dimension from 20 to 5 samples. The flattened output (160 features) is mapped to a 64-dimensional feature vector through a fully connected layer.

LSTM pathway architecture specifications

The TSM pathway utilizes a single-layer LSTM (input size = 6, hidden size = 16) to process 6-channel thermal measurements. Final hidden state (16 dimensions) is mapped to a 32-dimensional feature vector through linear transformation.

Fusion network and output generation

CNN (64 dimensions) and LSTM (32 dimensions) features are concatenated (96 dimensions total) and processed through two fully connected layer: 96 →32 dimensions with ReLU activations, followed by 32 →2 dimensions for simultaneous systolic and diastolic blood pressure prediction.

Training protocol and hyperparameter configuration

Model optimization employs Adam optimizer (learning rate 0.01) with adaptive learning rate scheduling (Reduced by 0.5, patience = 20 epochs). Batch size is 32 with 200 training epochs.

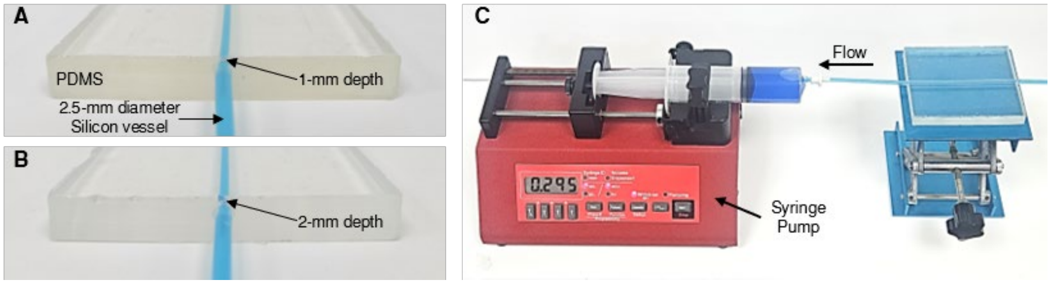

Fig. S1. Blood vessel phantom models and controlled flow experiment setting. (**A**) 1-mm vessel depth model. (**B**) 2-mm vessel depth model. (**C**) Controlled flow experimental setting using syringe pump.

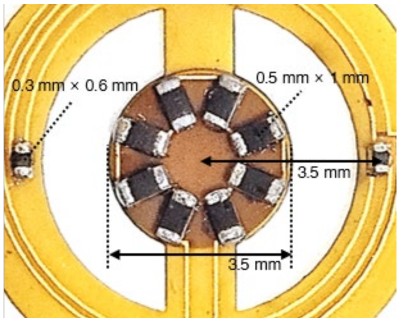

Fig. S2. Optical image of thermal actuator and thermistor.

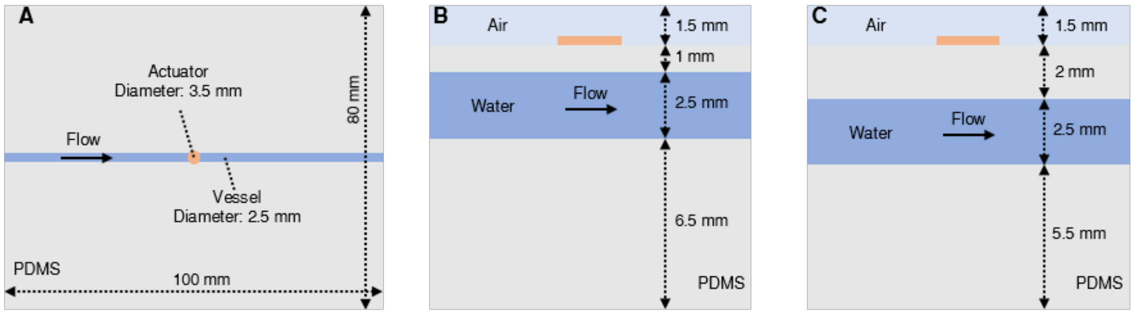

Fig. S3. Schematic illustrations of three-dimensional FEA modeling geometry with detailed dimensional specifications. (**A**) Top view of the computational domain (100 mm × 80 mm) showing the thermal actuator (3.5-mm diameter) positioned centrally and the embedded cylindrical vessel (2.5-mm diameter) relative to the flow direction. (**B**), (**C**) Cross-sectional side views for vessel depths of 1 mm (**B**) and 2 mm (**C**).

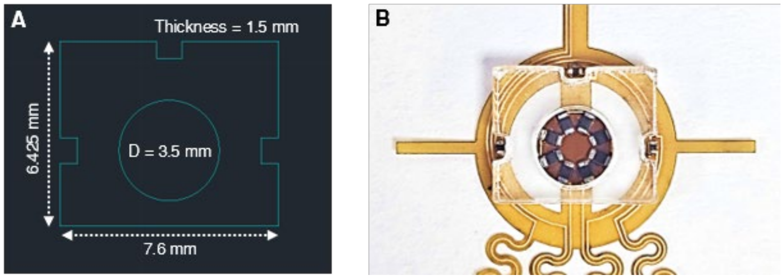

Fig. S4. Acrylic support specification. (**A**) AutoCAD 2022 design. (**B**) Laser cut 1.5-mm thickness support placed on fPCB

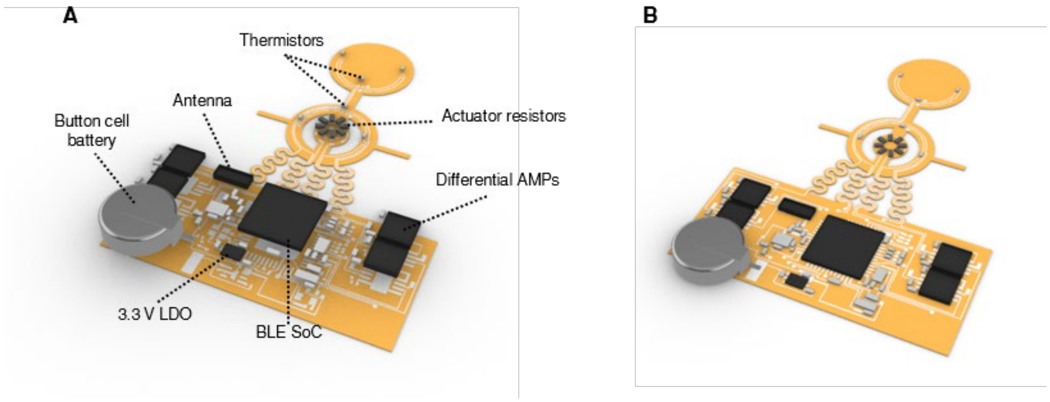

Fig. S5. Illustration of the wireless blood flow sensing platform. (**A**) Exploded-view illustration. (**B**) Components aligned.

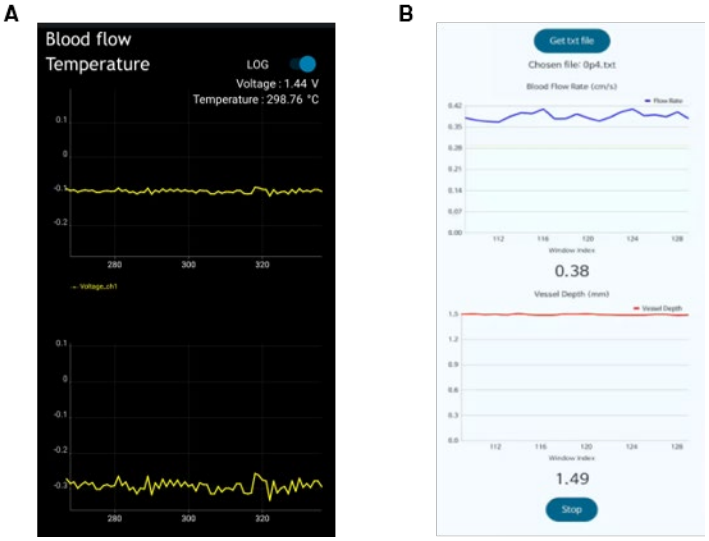

Fig. S6. Android application interfaces for data acquisition and real-time analysis. (**A**) Data logging interface for wireless sensor measurements. (**B**) Real-time blood flow rate and vessel depth prediction interface with deep learning inference.

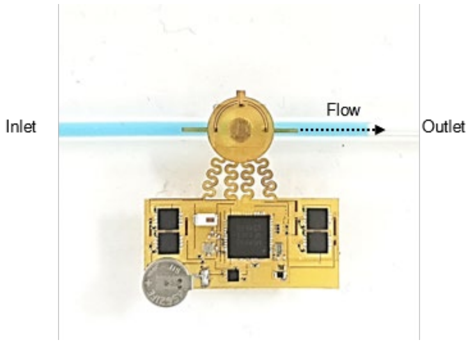

Fig. S7. An optical image of a wireless blood flow sensing platform on a PDMS vessel model for bench studies.

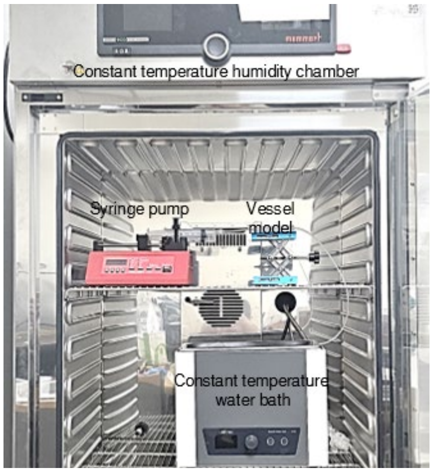

Fig. S8. Bench study settings (constant temperature humidity chamber, syringe pump, vessel model, constant temperature water bath).

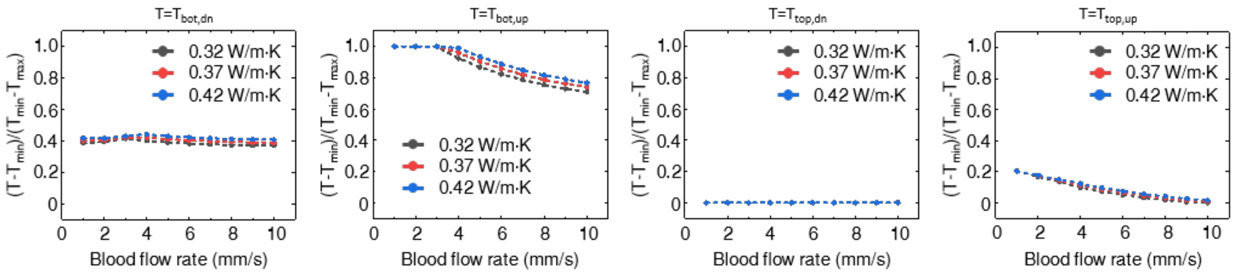

Fig. S9. Effect of skin thermal conductivity on normalized thermal sensing readings across different flow rates. T_{min} = min($T_{bot,dn}$, $T_{bot,up}$, $T_{top,dn}$, $T_{top,up}$, $T_{bot,ref}$, $T_{top,ref}$) and T_{max} = max($T_{bot,dn}$, $T_{bot,up}$, $T_{top,dn}$, $T_{top,up}$, $T_{bot,ref}$, $T_{top,ref}$)

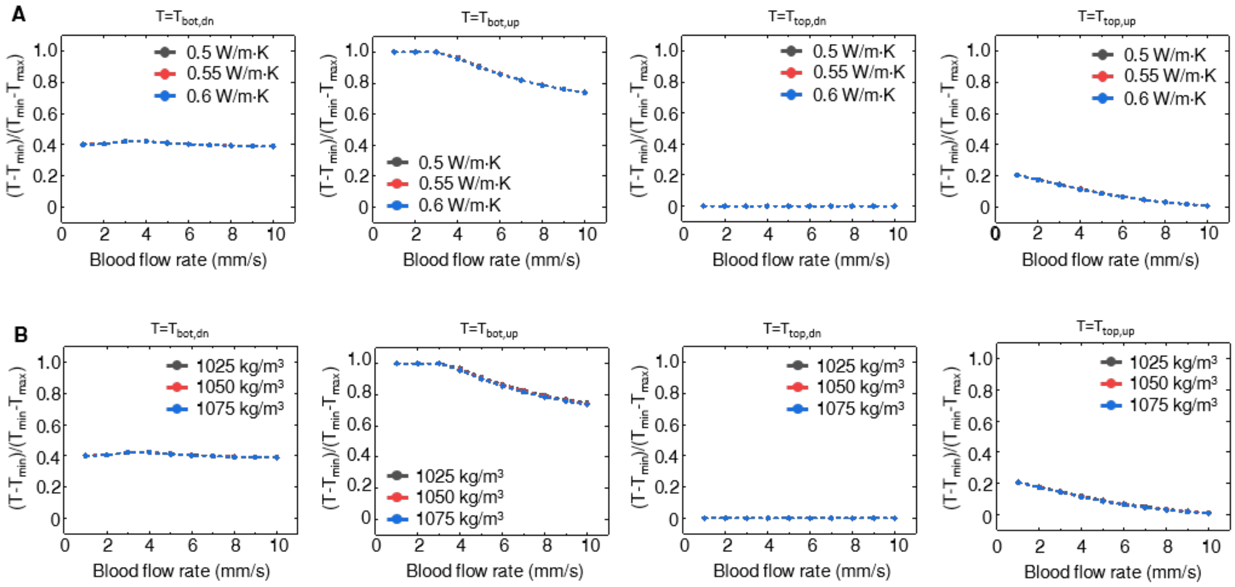

Fig. S10. Effect of blood thermal conductivity (A) and density (B) on normalized thermal sensing readings across different flow rates.

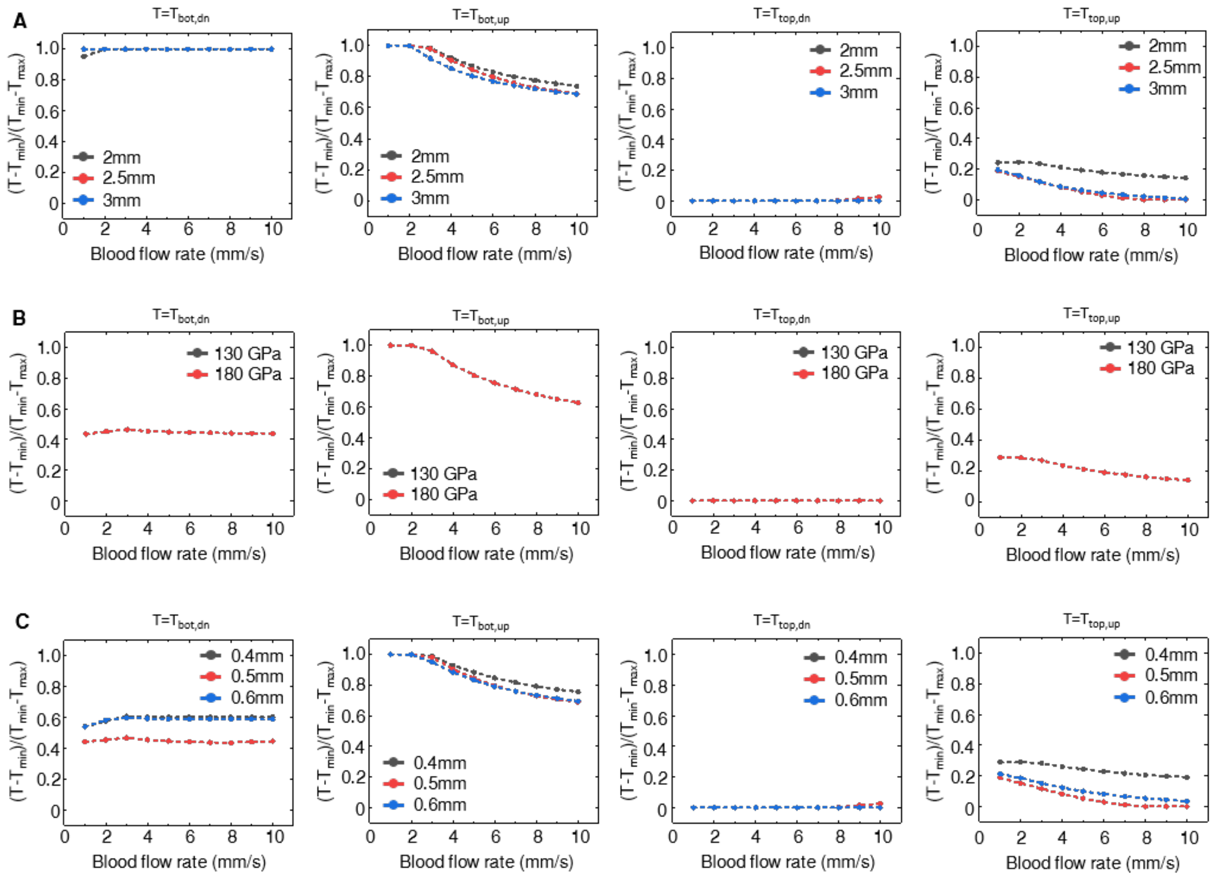

Fig. S11. Effect of vessel diameter (A), wall stiffness (B), and wall thickness (C) on normalized thermal sensing readings across different flow rates.

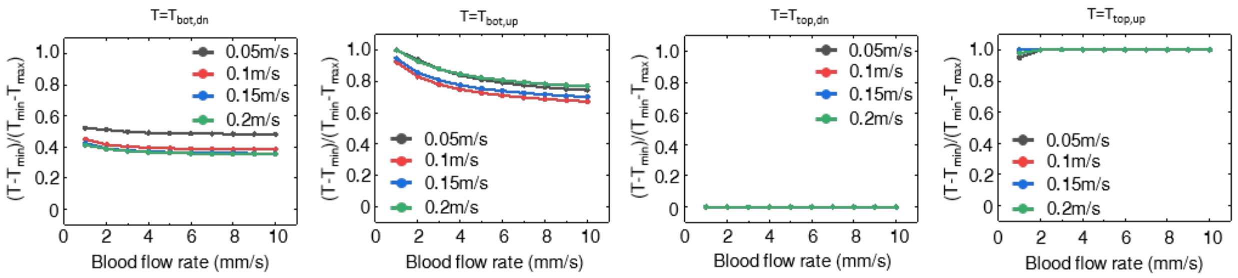

Fig. S12. Effect of ambient airflow on normalized thermal sensing readings across different flow rates.

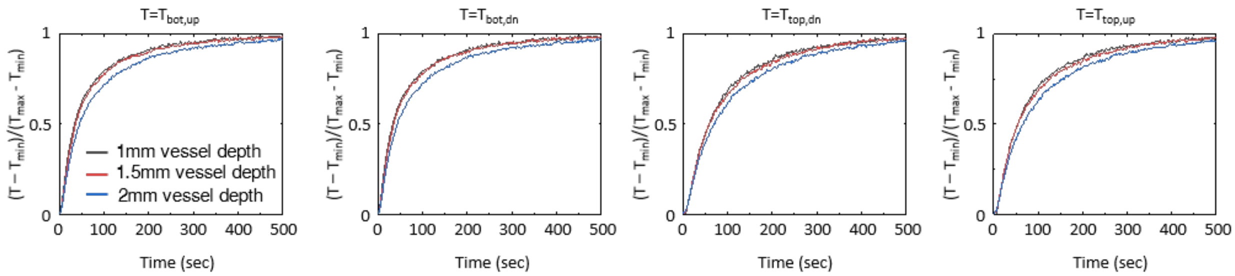

Fig. S13. Response characteristics of thermal sensors at different vessel depths.

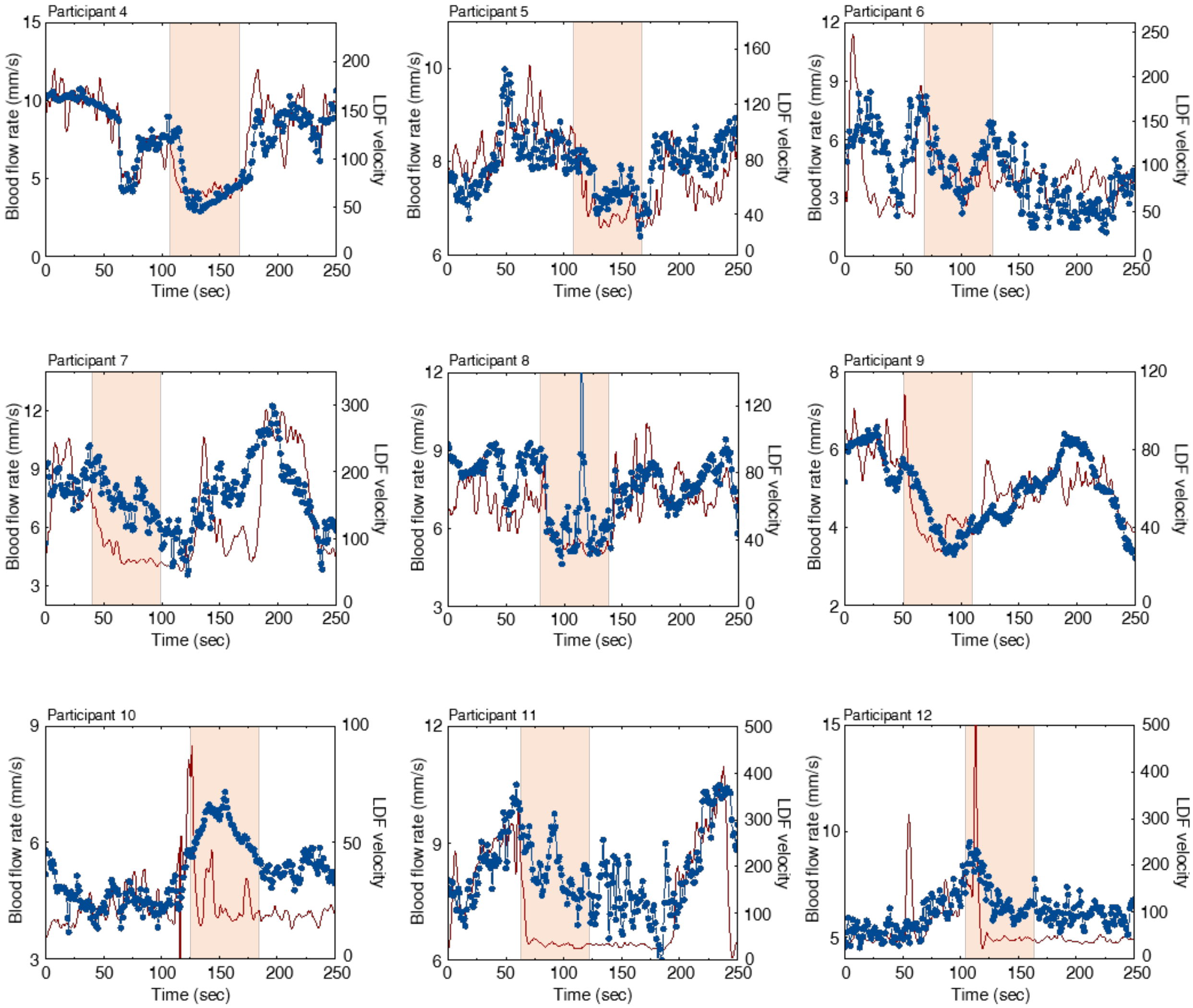

Fig. S14. Laser doppler flowmetry (LDF) results on participant 4-12. Blue markers and red lines indicate blood flow rate measured from our device and LDF velocity measurements, respectively. Highlighted regions represent external vascular compression using silicon wire.

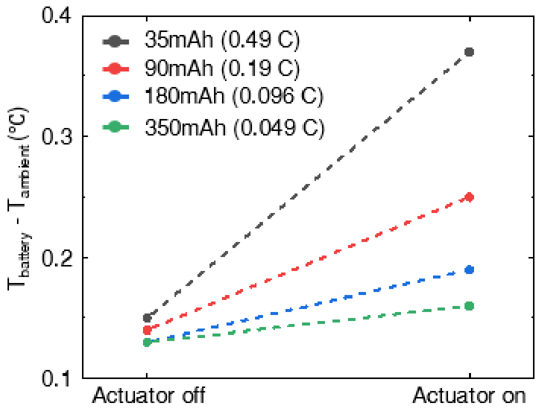

Fig. S15. Battery temperature elevation during device thermal actuation across different discharge rates. $T_{ambient}$ denotes the ambient temperature measured at a distance of 0.3 m from the device and maintained at 22 ± 0.5 °C throughout the experiment.

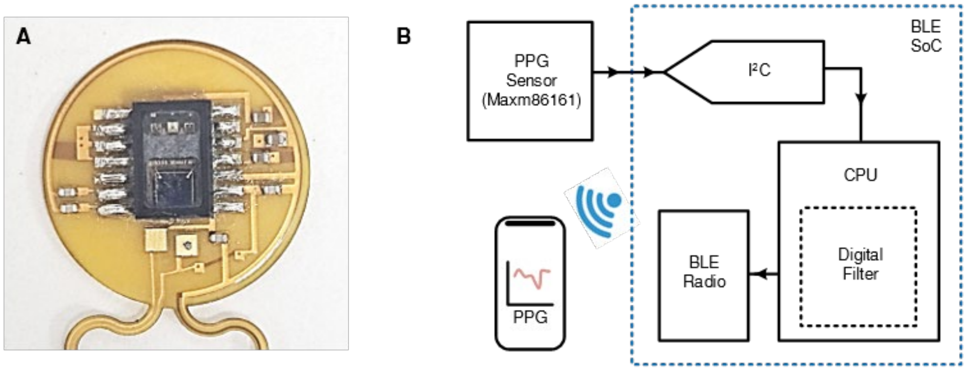

Fig. S16. PPG sensor configuration. (**A**) Optical image of Maxm86161. (**B**) schematic block diagram of PPG sensor

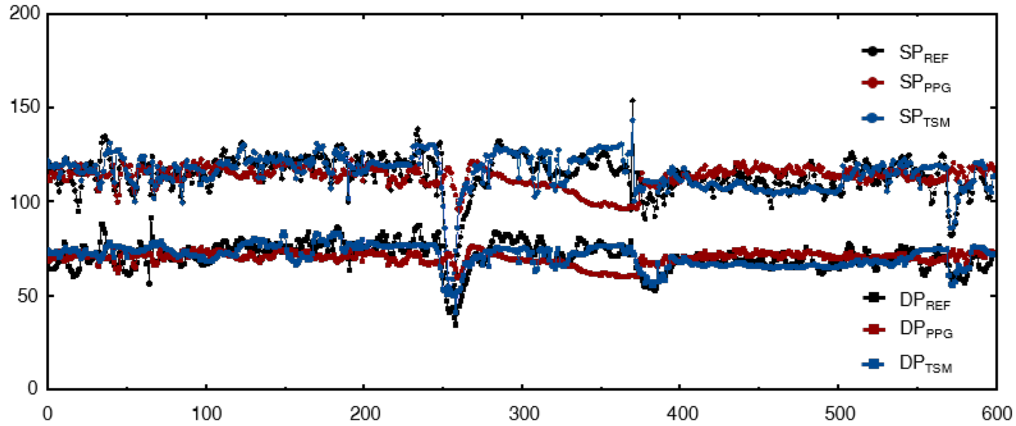

Fig. S17. Systolic and diastolic blood pressure during Valsalva maneuver.

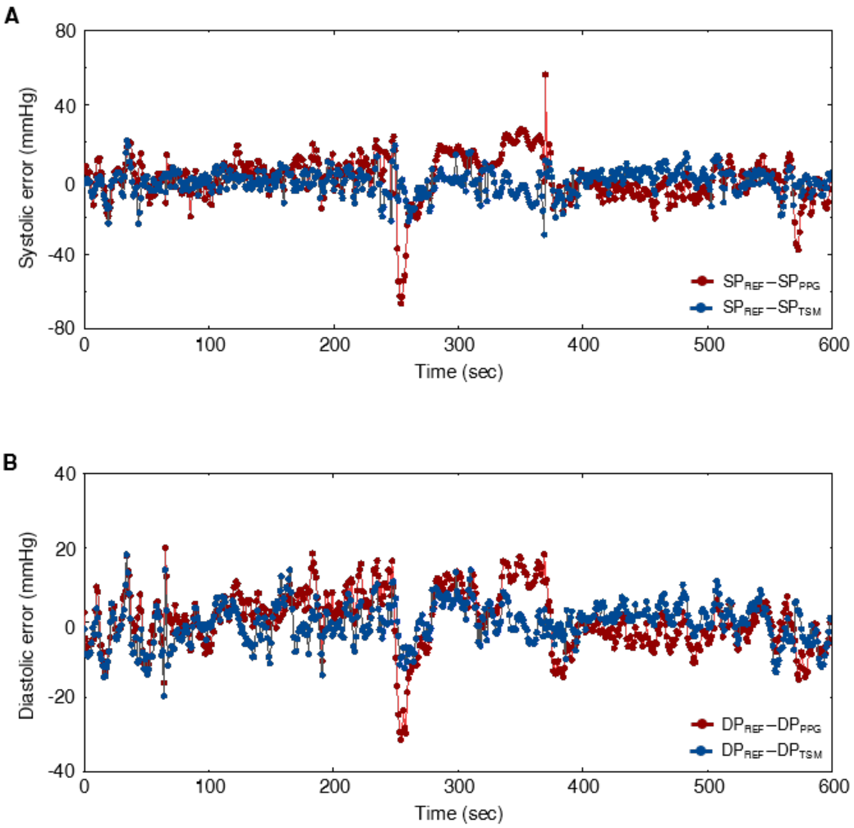

Fig. S18. Blood pressure prediction errors as a function of time. (A) Systolic pressure errors. (B) Diastolic pressure errors.

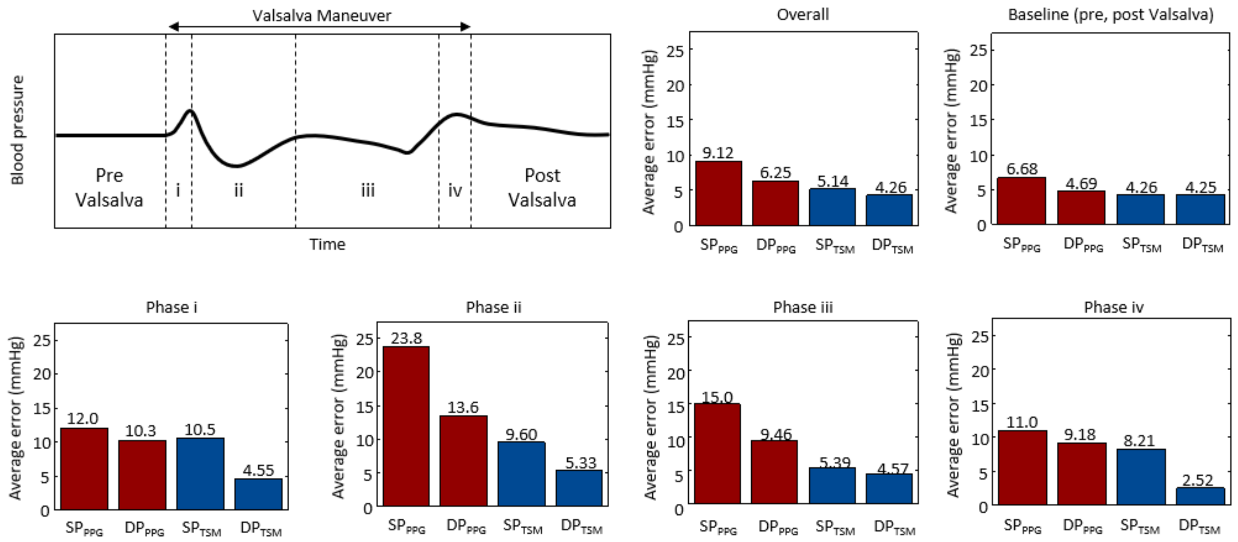

Fig. S19. Phase-specific blood pressure prediction errors.

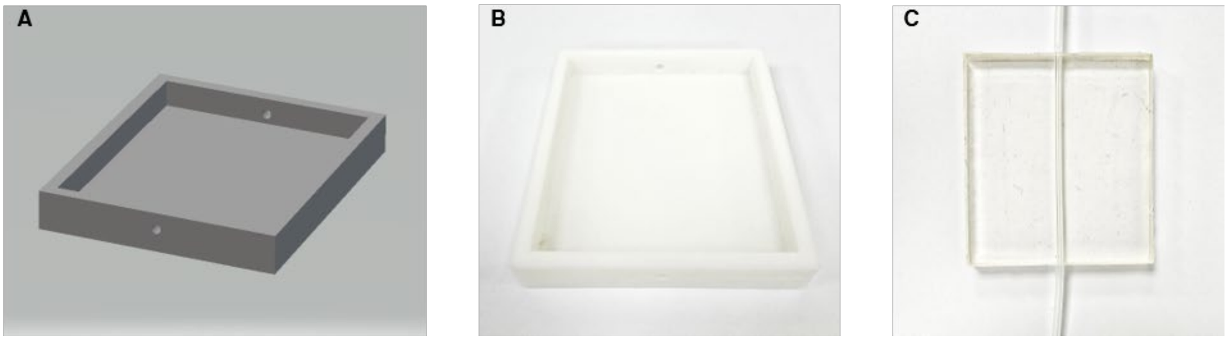

Fig. S20. PDMS vessel phantom fabrication. (**A**) Stereolithography(.stl) file. (**B**) 3D printed vessel phantom mold. (**C**) Finalized PDMS vessel phantom.

Specification	This work	Sci. Adv. 2015 [11]	Nat. Elec. 2021 [12]	Nat. Com. 2025 [13]
Sensor architecture	Multi-layer	Single-layer	Single-layer	Single-layer
Application	Blood flow & blood pressure	Blood flow	Sweat flow & biomarkers	Blood flow
Depth measurement capability	O (~2 mm)	O (~2 mm)	X	X
Measurement output	Continuous blood flow rate, continuous depth	Continuous blood flow rate, discrete depth	Continuous sweat flow rate	Flow classification (patent, low, occluded)
Accuracy	±0.12 mm/s (flow), ±0.07 mm (depth)	Qualitative (correlated with LSCI*)	~0.2 °C/μl/min sensitivity; error ~0.1-0.4 μl/min	Sensitivity/specificity for binary patency
Wireless capability	O (BLE)	X (wired)	O (BLE)	X (wired)

*Laser speckle contrast imaging

Table 1. Comparison and performance summary with prior works

Depth	Physiological interventions	Left wrist / Right wrist	Gender	Age	Height	Weight
Participant 1	Respiratory modulation	Left wrist	Male	22	179 cm	65.7 kg
Participant 2	External vascular compression	Right wrist	Male	26	180.2 cm	71.1 kg
Participant 3	Cycling exercise	Left wrist	Male	25	171.3 cm	68.5 kg

Table 2. Human subject information for perfusion index measurement

Depth	Left wrist / Right wrist	Gender	Age	Height	Weight	Fitzpatrick skin type(I-VI)
Participant 4	Left wrist	Female	26	160.2cm	49.7kg	III
Participant 5	Left wrist	Male	33	170.4cm	74.5kg	III
Participant 6	Left wrist	Female	25	158.2cm	50.1kg	II
Participant 7	Left wrist	Male	55	170cm	67.2kg	IV
Participant 8	Left wrist	Male	27	177.1cm	73kg	IV
Participant 9	Left wrist	Female	33	165cm	57.6kg	IV
Participant 10	Left wrist	Female	53	161cm	47.3kg	II
Participant 11	Left wrist	Male	26	180cm	70kg	III
Participant 12	Left wrist	Female	26	168.1cm	51.7kg	III

Table 3. Human subject information for LDF measurement.

Movie S1. Movie of real-time blood flow and vessel depth monitoring. Deep learning algorithm process thermal patterns and determine both blood flow and vessel depth simultaneously.

REFERENCES

1. K. Kisler, A. R. Nelson, A. Montagne, B. V. Zlokovic, Cerebral blood flow regulation and neurovascular dysfunction in Alzheimer disease. *Nat. Rev. Neurosci.* **18**, 419–434 (2017).

2. G. Eelen, P. De Zeeuw, M. Simons, P. Carmeliet, Endothelial cell metabolism in normal and diseased vasculature. *Circ. Res.* **116**, 1231–1244 (2015).

3. B. V. Zlokovic, R. F. Gottesman, K. E. Bernstein, S. Seshadri, A. McKee, H. Snyder, S. M. Greenberg, K. Yaffe, C. B. Schaffer, C. Yuan, T. M. Hughes, M. J. Daemen, J. D. Williamson, H. M. González, J. Schneider, C. L. Wellington, Z. S. Katusic, L. Stoeckel, J. I. Koenig, R. A. Corriveau, L. Fine, Z. S. Galis, J. Reis, J. D. Wright, J. Chen, Vascular contributions to cognitive impairment and dementia (VCID): A report from the 2018 National Heart, Lung, and Blood Institute and National Institute of Neurological Disorders and Stroke Workshop. *Alzheimers Dement.* **16**, 1714–1733 (2020).

4. "A discussion on the regulation of blood flow and pressure" in *Advances in Experimental Medicine and Biology* (Springer New York, 2016), pp. 129–135; http://link.springer.com/10.1007/978-1-4939-3023-4_16.

5. L. Sonnenschein, T. Etyang. Blood flow and health. *AJBSR* **22**, 374–392 (2024).

6. R. M. Bober, R. V. Milani, S. M. Kachur, D. P. Morin, Assessment of resting myocardial blood flow in regions of known transmural scar to confirm accuracy and precision of 3D cardiac positron emission tomography. *EJNMMI Res* **13**, 87 (2023).

7. B. Kerimkhan, A. Nedzved, A. Zhumadillayeva, K. Dyussekeyev, G. Uskenbayeva, B. Sultanova, L. Rzayeva, Automation of flow analysis in scleral vessels based on descriptive-associative algorithms. *Sci. Rep.* **13**, 4650 (2023).

8. M. K. Razavi, D. P. T. Flanigan, S. M. White, T. B. Rice, A real-time blood flow measurement device for patients with peripheral artery disease. *J. Vasc. Interv. Radiol.* **32**, 453–458 (2021).

9. P. Blanco, Volumetric blood flow measurement using Doppler ultrasound: Concerns about the technique. *J. Ultrasound* **18**, 201–204 (2015).

10. A. Oglat, M. Matjafri, N. Suardi, M. Oqlat, M. Abdelrahman, A. Oqlat, A review of medical doppler ultrasonography of blood flow in general and especially in common carotid artery. *J. Med. Ultrasound* **26**, 3 (2018).

11. R. C. Webb, Y. Ma, S. Krishnan, Y. Li, S. Yoon, X. Guo, X. Feng, Y. Shi, M. Seidel, N. H. Cho, J. Kurniawan, J. Ahad, N. Sheth, J. Kim, J. G. Taylor Vi, T. Darlington, K. Chang, W. Huang, J. Ayers, A. Gruebele, R. M. Pielak, M. J. Slepian, Y. Huang, A. M. Gorbach, J. A. Rogers, Epidermal devices for noninvasive, precise, and continuous mapping of macrovascular and microvascular blood flow. *Sci. Adv.* **1**, e1500701 (2015).

12. K. Kwon, J. U. Kim, Y. Deng, S. R. Krishnan, J. Choi, H. Jang, K. Lee, C.-J. Su, I. Yoo, Y. Wu, L. Lipschultz, J.-H. Kim, T. S. Chung, D. Wu, Y. Park, T. Kim, R. Ghaffari, S. Lee, Y. Huang, J. A. Rogers, An on-skin platform for wireless monitoring of flow rate, cumulative loss and temperature of sweat in real time. *Nat. Electron.* **4**, 302–312 (2021).

13. Y. Deng, H. M. Arafa, T. Yang, H. Albadawi, R. J. Fowl, Z. Zhang, V. Kandula, A. Ramesh, C. Correia, Y. Huang, R. Oklu, J. A. Rogers, A. S. Carlini, A soft thermal sensor for the continuous assessment of flow in vascular access. *Nat. Commun.* **16**, 38 (2025).

14. J. Fortin, D. E. Rogge, C. Fellner, D. Flotzinger, J. Grond, K. Lerche, B. Saugel, A novel art of continuous noninvasive blood pressure measurement. *Nat. Commun.* **12**, 1387 (2021).

15. E. M. Lee, When and how to use ambulatory blood pressure monitoring and home blood pressure monitoring for managing hypertension. *Clin. Hypertens.* **30**, 10 (2024).

16. A. Dadlani, K. Madan, J. P. S. Sawhney, Ambulatory blood pressure monitoring in clinical practice. *Indian Heart J.* **71**, 91–97 (2019).

17. Y. Degiorgis, M. Proença, Y. Ghamri, G. Hofmann, M. Lemay, P. Schoettker, Photoplethysmography-based blood pressure monitoring could improve patient outcome during anesthesia induction. *J. Pers. Med.* **12**, 1571 (2022).

18. D. Konstantinidis, P. Iliakis, F. Tatakis, K. Thomopoulos, K. Dimitriadis, D. Tousoulis, K. Tsioufis, Wearable blood pressure measurement devices and new approaches in hypertension management: The digital era. *J. Hum. Hypertens.* **36**, 945–951 (2022).

19. M. Elgendi, F. Haugg, R. R. Fletcher, J. Allen, H. Shin, A. Alian, C. Menon, Recommendations for evaluating photoplethysmography-based algorithms for blood pressure assessment. *Commun. Med.* **4**, 140 (2024).

20. C. Tantithamthavorn, S. McIntosh, A. E. Hassan, K. Matsumoto, An empirical comparison of model validation techniques for defect prediction models. *IIEEE Trans. Software Eng.* **43**, 1–18 (2017).

21. M. M. Elshal, A. M. Hasanin, M. Mostafa, R. M. Gamal, Plethysmographic peripheral perfusion index: Could it be a new vital sign? *Front. Med.* **8**, 651909 (2021).

22. K. Gupta, B. Rastogi, P. K. Gupta, M. K. Bansal, P. Kalra, S. Kaur, Perfusion index to assess hemodynamic changes and analgesia with premedication of fentanyl versus nalbuphine during general anesthesia. *AAN* **4**, 255–259 (2019).

23. A. Grunovas, E. Trinkunas, A. Buliuolis, E. Venskaityte, J. Poderys, K. Poderiene, Cardiovascular response to breath-holding explained by changes of the indices and their dynamic interactions. *Biol. Syst. Open Access* **05**, 1000152 (2015).

24. I. Taneja, M. S. Medow, D. A. Clarke, A. J. Ocon, J. M. Stewart, Postural change alters autonomic responses to breath-holding. *Clin. Auton. Res.* **20**, 65–72 (2010).

25. M. E. Moir, A. T. Corkery, K. B. Miller, A. G. Pearson, N. A. Loggie, A. A. Apfelbeck, A. J. Howery, J. N. Barnes, The independent and combined effects of aerobic exercise intensity and dose differentially increase post-exercise cerebral shear stress and blood flow. *Exp. Physiol.* **109**, 1796–1805 (2024).

26. S. K. Nyberg, O. K. Berg, J. Helgerud, E. Wang, Blood flow regulation and oxygen uptake during high-intensity forearm exercise. *J. Appl. Physiol.* **122**, 907–917 (2017).

27. M. T. Chua, A. Sim, S. F. Burns, Acute and chronic effects of blood flow restricted high-intensity interval training: A systematic review. *Sports Med. Open* **8**, 122 (2022).

28. A. Bergholz, G. Greiwe, K. Kouz, B. Saugel, Continuous blood pressure monitoring in patients having surgery: A narrative review. *Medicina* **59**, 1299 (2023).

29. R. Mukkamala, J.-O. Hahn, O. T. Inan, L. K. Mestha, C.-S. Kim, H. Toreyin, S. Kyal, Toward ubiquitous blood pressure monitoring via pulse transit time: Theory and practice. *IEEE Trans. Biomed. Eng.* **62**, 1879–1901 (2015).

30. M. Komolova, M. A. Adams, Moment-to-moment characteristics of the relationship between arterial pressure and renal interstitial hydrostatic pressure. *Hypertension* **56**, 650–657 (2010).

31. J. Perdereau, T. Chamoux, E. Gayat, A. Le Gall, F. Vallée, J. Cartailler, J. Joachim, Blood pressure estimation using explainable deep-learning models based on photoplethysmography. *Anesth. Analg.* **140**, 119–128 (2025).

32. D. U. Jeong, K. M. Lim, Combined deep CNN–LSTM network-based multitasking learning architecture for noninvasive continuous blood pressure estimation using difference in ECG-PPG features. *Sci. Rep.* **11**, 13539 (2021).

33. B. Kamanditya, Y. N. Fuadah, N. Q. Mahardika T., K. M. Lim, Continuous blood pressure prediction system using Conv-LSTM network on hybrid latent features of photoplethysmogram (PPG) and electrocardiogram (ECG) signals. *Sci. Rep.* **14**, 16450 (2024).

34. Y. Park, H. Luan, K. Kwon, T. S. Chung, S. Oh, J.-Y. Yoo, G. Chung, J. Kim, S. Kim, S. S. Kwak, J. Choi, H.-P. Phan, S. Yoo, H. Jeong, J. Shin, S. M. Won, H.-J. Yoon, Y. H. Jung, J. A. Rogers, Soft, full Wheatstone bridge 3D pressure sensors for cardiovascular monitoring. *NPJ Flex. Electron* **8**, 6 (2024).

35. D. S. Goldstein, W. P. Cheshire, Beat-to-beat blood pressure and heart rate responses to the Valsalva maneuver. *Clin. Auton. Res.* **27**, 361–367 (2017).

36. F. Pedregosa, G. Varoquaux, N. Org, A. Gramfort, V. Michel, B. Thirion, O. Grisel, M. Blondel, P. Prettenhofer, R. Weiss, V. Dubourg, J. Vanderplas, A. Passos, D. Cournapeau, M. Brucher, M. Perrot, E. Duchesnay, Scikit-learn: Machine learning in python. *J. Mach. Learn. Res.* **18**, 2825–2830 (2011).
